# Program Theory and Core Outcome Set Development for a Technology-Assisted Counseling Intervention in Dementia: Multimethods Study

**DOI:** 10.2196/81669

**Published:** 2026-01-20

**Authors:** Dorothee Bauernschmidt, Anja Bieber, Ronja Hubrich, Janina Wittmann, Gabriele Meyer

**Affiliations:** 1 Institute of Health, Midwifery and Nursing Science, Medical Faculty, University Medicine Halle, Martin Luther University Halle-Wittenberg, Magdeburger Straße 8, Halle (Saale), 06112, Germany, 49 3455574466

**Keywords:** theory, core outcome set, logic model, counseling, family caregiver, dementia

## Abstract

**Background:**

Counseling in family dementia care aims to support caregivers in mastering challenges. The use of information and communication technologies (ICT) to administer counseling can improve accessibility. Evidence syntheses report inconsistent findings on the effectiveness of technology-assisted counseling. There is a considerable heterogeneity in outcomes assessed in clinical trials, and approaches to develop and evaluate interventions are not guided by theory in most cases.

**Objective:**

This study aims to develop an initial program theory of a technology-assisted counseling intervention for family dementia caregivers and to create the data basis for the consensus process of a core outcome set.

**Methods:**

We integrated the methodological strands for the development of a program theory and a core outcome set in an innovative way. A scoping review was conducted to collect data on characteristics and theoretical foundations of technology-mediated counseling interventions as well as outcomes of clinical studies. We explored the lived experience of relevant interest-holders and conducted semistructured interviews applying a phenomenological approach to data analysis. Synthesis of findings was performed by developing a logic model and formulating an initial program theory.

**Results:**

We included 69 records reporting on 34 interventions. Designs and other study characteristics vary, and interventions are heterogeneous in terms of components and ICT used for delivering counseling. We conducted interviews with 15 family caregivers and 12 counselors. The themes *being affected*, *feeling insecure and helpless in the face of the health care system*, and *search for information and communicative exchange* illustrate the caregivers’ lifeworld perception. Themes identified in counselors’ interviews comprise *work attitude and standards*, *unpredictability*, *expectations*, *working conditions*, *organizational influence*, and *tools: techniques and networking*. The constitutive pattern of *having/being somebody to count on* was incorporated into the program theory. In the theory of change, we describe the way to a sustainable supportive cooperation between caregivers and counselors ensuring ongoing support throughout the caregiving process. We explicate the effects of the technology-assisted counseling intervention such as improved knowledge, attitude, and interaction, as well as stability and safety of care in the outcomes chain. The theory of action comprises the inputs, activities, and outputs of the intervention. The graphical synthesis of findings is presented in the logic model.

**Conclusions:**

To effectively develop, implement, and evaluate technology-assisted counseling in family dementia care, a theory-led approach is essential. A carefully modeled intervention that combines technological options with in-person counseling may help to overcome disparities in access to health care and improve accessibility to counseling. A supportive working environment for counselors, in which artificial intelligence is used to reduce time spent on documentation and administrative tasks, may help mitigate the effects of the growing shortage of skilled professionals.

## Introduction

Counseling interventions in family dementia care aim to support caregivers in mastering challenges in caregiving. Information and communication technologies (ICT) are used to deliver counseling in an easily accessible way [1-3].

Counseling may contribute to mitigating the negative impact family dementia care can exert on caregivers, which is described in terms of burden, depression, decreased health and quality of life, as well as social isolation [4,5]. In light of the predicted increase in the number of persons with dementia [6], the need for support interventions for family caregivers is also likely to increase. Provided by professionals, counseling can be defined as the “use of an interactive helping process focusing on the needs, problems, or feelings of the patient and significant others to enhance or support coping, problem-solving, and interpersonal relationships” [7]. The use of ICT is discussed to reduce barriers to utilization by overcoming distances, enabling persons who are homebound or living in rural areas to participate in services, offering anonymous counseling, or fostering asynchronous communication beneficial for persons who are employed [3,8]. Types of ICT have expanded over time: long-established helpline services use the telephone as a widespread and undemanding technology. More recently, counseling is provided via videoconferencing software, email, or chats using mobile devices and applications, thus leading to increased demands on technological infrastructure, equipment, and digital literacy [8].

Technology-assisted counseling (also referred to as “technology-based” in previous publications) is a complex intervention [9], and the development, implementation, and evaluation of complex interventions are challenging. The effectiveness of technology-assisted counseling in dementia has not yet been proven: Evidence syntheses of technology-assisted psychosocial interventions including counseling for caregivers of persons with dementia show inconsistent findings on positive effects on outcomes such as burden, depression, or quality of life [1-3,10]. Our own meta-analyses revealed no significant effects of technology-assisted counseling on depressive symptoms, burden, and self-efficacy or mastery perceived by family caregivers of persons with dementia [11]. We found a considerable heterogeneity in outcomes and a lack of theoretical approaches guiding the development, implementation, and evaluation of the interventions [8,11].

Therefore, we conducted the ProCOS project: “Development and Evaluation of a Technology-Assisted Counseling Intervention for Family Caregivers of Persons With Dementia – Program Theory and Preparation of a Core Outcome Set” [12]. Within the 12-month project, we aimed at creating the foundation for the future consensus process of a core outcome set (COS) and at developing a program theory by combining the methodological strands of the two developmental processes in an innovative way.

A COS is “an agreed standardized collection of outcomes which should be measured and reported, as a minimum, in all trials for a specific clinical area” [13]. The use of a COS reduces heterogeneity in clinical trials and enhances comparability and thus synthesis of evidence [13]. Core outcome sets for the evaluation of health care interventions and of psychosocial community-based interventions for persons with dementia living at home predominately focus on outcomes of persons with dementia [14,15]. A set of measures has been recommended to evaluate a broad range of psychosocial interventions for persons with dementia and their family caregivers [16]. There is no COS that specifically focuses on technology-assisted counseling interventions for family dementia caregivers.

The Framework for Developing and Evaluating Complex Interventions identifies program theory as a core element of complex interventions [9]. This underlines the importance of theoretical approaches to successfully develop, implement, and evaluate complex interventions. A program theory is an “explicit theory of how an intervention is understood to contribute to its intended or observed outcomes” [17]. Following the approach introduced by Funnell and Rogers [17], we developed a “purposeful program theory” comprising the theory of change, the outcomes chain, and the theory of action. The theory of change explicates the central mechanism of how the intended changes can be achieved, and the theory of action explains how the intervention is designed to initiate the theory of change. These elements are linked by the outcomes chain comprising the immediate and intermediate outcomes and the impact of the intervention, as well as hypothesized relationships between outcomes [17]. In line with the updated UK Medical Research Council framework, we understand a program theory as a detailed textual description [18]. Logic models serve as visual representations of program theories [17,18]. Detailed logic models graphically illustrate the (assumed) causal mechanisms through which an intervention is expected to produce outcomes, as well as contextual dependencies and preconditions [18]. Developing a program theory that incorporates perspectives of diverse interest-holders and integrates theoretical and empirical knowledge at the beginning of interventional research is considered best practice [9]. Program theories and logic models are adapted and refined throughout the development, implementation, and evaluation of complex interventions to address the question, “What works in which circumstances and how?”—thus applying a theory-based approach [9].

As the methodological strands both for developing a program theory and a COS integrate knowledge obtained from literature and perspectives from different interest-holders [13,17], we brought these two processes together to develop a program theory and prepare the consensus process of a COS of a technology-assisted counseling intervention for caregivers of persons with dementia. The central instrument for this innovative approach is the logic model. As graphical representations of program theories [17], logic models have been used to synthesize data [19,20] and to visualize assumed causal relationships and mechanisms of action of complex interventions [21]. By fostering a shared understanding among interest-holders [9,17], we used the logic model for data synthesis, and we will integrate it in the future consensus process of the COS, allowing interest-holders to critically review the quality of the program theory. To our knowledge, this approach has not yet been implemented.

By drawing on previous work on the effectiveness and implementation success of technology-assisted counseling in dementia [8,11,22], we therefore aimed at addressing gaps in knowledge. Thus, our first objective was to develop a program theory of a technology-assisted counseling intervention for family dementia caregivers. Our secondary objective was to compile lists of (potential) outcomes that have been identified by caregivers or counselors or assessed in clinical studies.

The following two sets of questions guided our research:

1. What interventions that use ICT to provide counseling for family dementia caregivers are described in literature? What are the characteristics of these interventions? What theoretical underpinnings for intervention development and implementation are explicated in the form of theoretical references, program theories, or logic models? What outcomes have been examined in clinical trials? What assessment instruments have been used?
2. How do family caregivers for persons with dementia and counselors experience counseling services? What expectations do persons seeking or providing counseling have of each other? Which outcomes should or could be achieved through counseling, and how can these outcomes be achieved? Which factors have an impact on the effectiveness of counseling? What are possible outcomes for assessing the effectiveness of counseling interventions?

## Methods

The ProCOS project was registered with the Core Outcome Measures in Effectiveness Trials (COMET) Initiative [23]. The study protocol has been published [12].

### Study Design

We adopted a multimethod design including a literature review and a qualitative substudy [24]. Results were graphically synthesized into a logic model and an initial program theory was formulated comprising the theory of change, the outcomes chain, and the theory of action [17].

We applied the PRISMA-ScR (Preferred Reporting Items for Systematic Reviews and Meta-Analyses, Extension for Scoping Reviews) [25], the SRQR (Standards for Reporting Qualitative Research) [26], and the COS-STAR (Core Outcome Set—Standards for Reporting) [27] to structure our report.

### Scoping Review

We aimed to map the knowledge on technology-assisted counseling interventions for family caregivers of persons with dementia and followed the Joanna Briggs Institute methodological guidance for scoping reviews [28].

#### Eligibility Criteria

We included studies on interventions using ICT to deliver individualized counseling to caregivers of persons with dementia. Publications in the German or English language were accepted, irrespective of their design. Detailed inclusion and exclusion criteria according to the Population-Concept-Context scheme are provided in the study protocol [12].

#### Search Strategy and Information Sources

By updating the literature search of a previous systematic review [8,11,22], we searched the databases CINAHL, MEDLINE via PubMed, Cochrane Library, and PsycINFO (December 2023) and conducted additional forward and backward citation searching as well as a free web search (April 2024). The database-specific search strategies and the search terms of the free web search are provided in Multimedia Appendix 1.

#### Selection of Sources of Evidence

Titles, abstracts, and full texts were screened independently by two researchers (DB and RH) using the Rayyan web app [29]. Discrepancies in decisions were resolved by discussion.

#### Data Charting Process and Data Items

We extended a previously developed data extraction sheet and extracted data on study characteristics (year of publication, country of study conduct, design and methods, and number of participants) and on outcomes examined as well as assessment instruments used in included studies. We also applied criteria from the Template for Intervention Description and Replication (TIDieR) checklist [30] and from the revised Criteria for Reporting the Development and Evaluation of Complex Interventions (CReDECI 2) guideline [31] in order to collect information on objectives, components, theoretical underpinnings of counseling interventions, technology and materials used for delivering counseling, frequency and duration of sessions, and implementation issues. Data extraction was performed by one reviewer (DB). A cross-check of extracted data was conducted by another researcher (RH) for 20% of included interventions indicating accuracy and completeness of data extraction.

### Qualitative Substudy

With the aim to explore the lived experience of counseling in dementia care, we conducted interviews with interest-holders in caregiving and counseling in the context of family dementia care.

#### Qualitative Approach and Research Paradigm

We adopted a phenomenological perspective [32] and focused on the lifeworld experiences of caregivers and counselors.

#### Context, Researcher Characteristics, and Reflexivity

All interviews and a preliminary analysis were conducted by the primary investigator (DB), who is experienced in phenomenological research. Results were then discussed within the research team. Team members are nursing scientists with extensive experience in nursing practice and dementia research, and a physiotherapist.

#### Units of Study and Sampling Strategy

Interviews were conducted with family caregivers and counselors. We used a purposive sampling strategy [33] to obtain a heterogeneous sample. Predefined criteria for recruitment of dementia caregivers were age, gender, socioeconomic status, and family relationship, as well as spatial and emotional proximity to the person receiving care. Predefined criteria for recruitment of counselors were disciplinary background, professional qualifications, duration of counseling experience, and characteristics of employing organizations. We included persons who have received or delivered counseling via technology, in-person, or both. While our efforts to recruit a heterogeneous sample of family dementia caregivers had limited success, we succeeded more in engaging counselors with diverse characteristics.

Invitations to participate and study information were distributed via existing contacts and networks established through previous research projects and the State Competence Center for Dementia of the German federal state of Saxony-Anhalt. In addition, we contacted counseling services and self-help organizations of family caregivers throughout Germany. Recruitment was completed when no new information emerged from interviews indicating that data saturation was achieved [34,35].

#### Data Collection Methods and Data Processing

We performed semistructured interviews using an interview guide with open-ended questions to give interviewees room to share their experiences [36]. Questions focusing on experiences in receiving and providing counseling were asked in the course of the interview. Finally, additional data on sociodemographic characteristics, information on the care arrangement, or on the professional situation were collected. The translated interview guide is provided in Multimedia Appendix 2.

Interviews were arranged at participants’ convenience to minimize time exposure and burden for participants. We planned to conduct individual interviews, but switched to group interviews at the request of some participating caregivers and counselors.

Recordings of interviews were transcribed verbatim using f4 transcription software [37]. Transcripts were checked for accuracy and pseudonymized by one researcher (RH).

#### Data Analysis

We conducted an interpretive phenomenological analysis applying the following modified working steps described by Diekelmann [38]:

1. The transcripts are read several times to obtain an overall understanding.
2. An interpretive summary of each interview is written.
3. Interpretive summaries are analyzed and discussed to identify emerging themes.
4. Disagreements in interpretation are resolved by returning to the text.
5. Through comparing and contrasting texts, the themes that recurred and reflected the shared practices and common meanings are identified and described.
6. As themes are compared, a constitutive pattern emerges that links the themes and is present in all interviews.
7. The themes and the constitutive pattern are described using quotations to illustrate findings.

Themes and the constitutive pattern illuminate the lifeworld perception of receiving or providing counseling in the context of family dementia care.

In addition, outcomes designated by caregivers and counselors were independently extracted from the transcripts by two researchers (DB and JW) and discussed intensively in order to include them as accessible statements [39] into the future consensus-building process.

### Synthesis of Data Through Logic Model Development and Formulation of a Preliminary Program Theory

Contrary to the original plan to conduct the data synthesis into the logic model and the formulation of the program theory consecutively, we brought these two working steps together in an iterative process. We switched back and forth between writing memos and graphically synthesizing the data. Following the recommendation made by Funnell and Rogers [17], we combined the inductive and deductive approach with articulating interest-holders’ mental models to develop the program theory. Mental models are diverse interest-holders’ beliefs about how a program achieves its results [17]. We integrated the mental models explicated by caregivers and counselors with data derived from literature and with information on programs operating in practice. We treated data extracted from studies included in the scoping review as qualitative data [20]. Data were charted and categorized [20], and assigned to the elements of the program theory [17].

The starting point for the development of the theory of change was the situation analysis for which we applied the guiding questions formulated by Funnell and Rogers [17]. Key aspects of the situation analysis are depicted as macro-, meso-, and microcontext in the logic model.

We then explicate our assumptions of how the intended changes can be achieved by formulating the theory of change: (hypothesized) mechanisms of effective counseling, as described by interview partners or identified in literature, were incorporated into the theory of change.

Outcomes derived from clinical studies or mentioned by participants were summarized in tables and served as the foundation of the outcomes chain. The development of the outcomes chain followed the steps outlined by Funnell and Rogers [17]: (1) listing of possible outcomes, (2) clustering outcomes and assigning working labels to each cluster, (3) arranging outcomes in a chain of if-then statements, (4) identifying feedback loops, and (5) validating the outcomes chain.

By explicating how the intervention is designed to initiate the theory of change, we developed the theory of action and identified resources (inputs), activities, and outputs [17]. We incorporated intervention components and strategies that were reported in the literature or by study participants as helpful or effective in achieving the intended outcomes in the theory of action.

### Patient and Public Involvement

We established a study advisory board consisting of a person with long-term experience in caring for family members with dementia and engagement in an informal support network for family caregivers, a person with extensive counseling experience, and an experienced researcher in the field of dementia care. Representing different groups of interest-holders, the members of the study advisory board critically reviewed the instruments and approaches for data collection and were involved in the discussion of results and conclusions for the design of a technology-assisted counseling intervention. Three virtual meetings were held in addition to feedback provided via email over the course of the 12-month project.

### Techniques to Enhance Trustworthiness

We applied the strategies of expert consultation and peer debriefing by consulting the members of the study advisory board throughout the research process.

### Ethical Considerations

The ethics committee of the Medical Faculty of the Martin Luther University Halle-Wittenberg approved the ProCOS study (no. 2023‐093). Persons interested in participating received written information on procedures prior to the interview. The time and place of the interviews were determined based on the participants’ preferences. We obtained written informed consent from the participants who were informed that the consent to participate can be withdrawn at any time. We maintained the security of data by storing data protected from access by persons who are not involved in the project. Audio recordings of interviews were pseudonymized during the transcription process. Study participants received a small gift (equivalent to US $5), but no financial compensation.

## Results

### Scoping Review

#### Selection of Sources of Evidence

**Figure 1.**
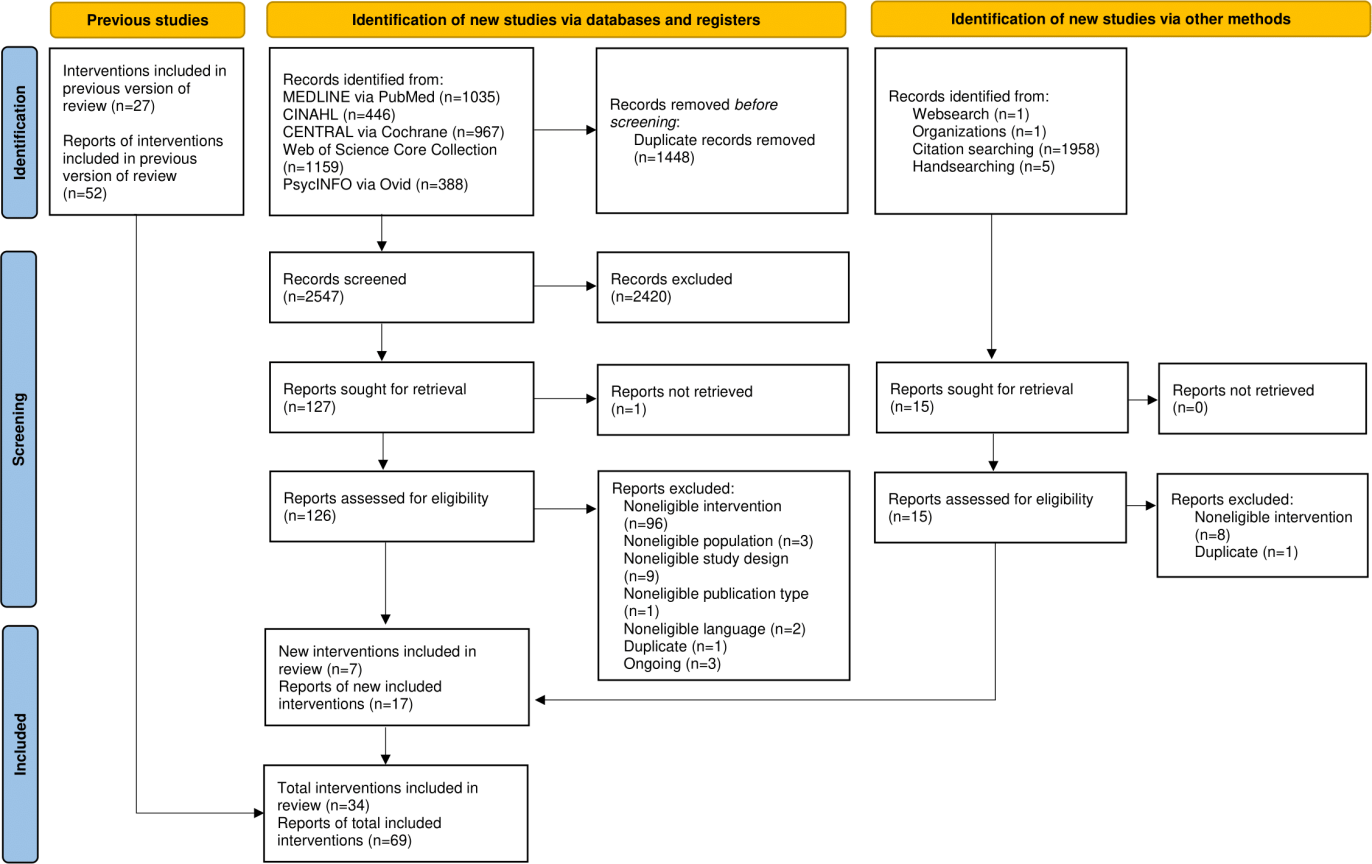
PRISMA-ScR (Preferred Reporting Items for Systematic Reviews and Meta-Analyses, Extension for Scoping Reviews) flow diagram for updated reviews [40].

The updated database search yielded 3995 records. An additional 1965 records were identified through complementary search strategies. After the removal of 1448 duplicates of reports identified via database search and 332 duplicates of reports identified via other methods, 4180 titles and abstracts were screened. A total of 141 full texts were screened for eligibility. Of these, 17 records reporting on 7 interventions were included. These new studies were combined with 52 records on 27 interventions from the previous review, resulting in 69 records reporting on 34 interventions that were included in the scoping review (Figure 1).

#### Characteristics of Sources of Evidence

The studies were published between 1993 and 2024 (last search April 2024) and were conducted in 10 countries (Australia, Canada, Germany, Israel, Italy, Japan, the Netherlands, Sweden, the United Kingdom, and the United States).

As we included studies irrespective of their design, topics, and methodological approaches varied. The effectiveness of 7 interventions on outcomes such as depressive symptoms, burden, and self-efficacy (refer to Table 1) has been evaluated by randomized controlled trials (RCTs) [41-63], including a small-scale RCT in the context of a pilot and feasibility study [64,65]. Nonrandomized trials [66-68] and a pre-post-intervention trial [69] assessed the effects on caregivers’ ability in disease management, burden, or mental health; quantitative descriptive approaches were conducted to examine sociodemographic characteristics of service users, reasons for use and topics discussed, advice provided, and satisfaction with services, as well as ways of access to counseling [70-82]. Qualitative approaches were used to explore expectations and experiences of caregivers with technology-assisted counseling services [59,65,70,83-92]. Case studies [93-95] examined individual counseling processes reflecting on the complexity of delivering counseling via ICT, as well as skills and knowledge needed to provide effective support. We included two process evaluation studies focusing on implementation issues when linking eHealth interventions to existing support [96] and combining a cognitive rehabilitation program with education and counseling [97]. Mixed methods approaches were applied to achieve an in-depth understanding of how the intervention works, to assess usability, and to describe and explain (non-)usage of services [56,57,98-103]. Detailed information on included references is presented in Multimedia Appendix 3 [8,30,31,41-109].

**Table 1.** Outcomes identified in the literature.

Outcomes and assessment instruments	Intervention^a^	References
Outcomes for caregivers		
Depression		
Zung Self-Rating Depression Scale	Coyne^a^ comparator or experimental	[42]
Center for Epidemiology Studies Depression Scale (CES-D)	FITT-C^b^	[44-50]
	RCTM^c^	[54-60,64,65]
Center for Epidemiologic Studies Depression Scale (CES-D-10)	CuidaTEXT	[98,104-106]
Center for Epidemiologic Studies Depression Scale–Revised (CESD-R)	TeleFAMILIES^d^	[69]
Geriatric Depression Scale (GDS)	FITT-D^e^	[43]
Mood Assessment Scale (MAD)	RCTM^c^	[54-60]
Grief		
Caregiver Grief Scale (CGS)	RCTM^c^	[64,65]
Guilt		
Caregiver Guilt Questionnaire (CGQ)	RCTM^c^	[64,65]
Anxiety		
Geriatric Anxiety Inventory (GAI)	RCTM^c^	[64,65]
Burden or caregiver distress		
Zarit Burden Interview (ZBI)	Coyne^a^ comparator or experimental	[42]
	FITT-C^b^	[44-50]
	FITT-D^e^	[43]
	TeleFAMILIES^d^	[69]
Zarit Burden Interview (ZBI-6)	CuidaTEXT	[98,104-106]
Burden Scale for Family Caregivers (BSFC)	ICSS^f^	[90-92,99-101]
Caregiver Burden Inventory (CBI)	Natale^a^	[66]
	De Cola^a^	[82]
Subjective stress		
Not specified (7-item measure of care-related strain; 1-item measure assessing the caregiver’s difficulty with the relative’s mental or emotional state); Adapted Zarit Burden Interview (ZBI)	RCTM^c^	[54-60]
Perceived stress		
Perceived Stress Scale (PSS)	RCTM^c^	[64,65]
Caregiver strain		
Modified Caregiver Strain Index (CSI)	CuidaTEXT	[98,104-106]
Reaction to care-receiver behavior		
Revised Memory and Behavior Problem Checklist (RMBPC)	FITT-C^b^	[44-50]
	FITT-D^e^	[43]
	TeleFAMILIES^d^	[69]
Emotional impact of neuropsychiatric symptoms		
Neuropsychiatric Inventory (NPI) Burden subscale	Dementelcoach	[61-63,67,68,96]
Upset		
Caregiver Behavioral Occurrence and Upset Scale (modeled on the Agitated Behavior in Dementia Scale)	Laver^a^	[51-53]
Behavioral symptom severity–distress		
Neuropsychiatric Inventory–Questionnaire (NPI-Q) Distress	CuidaTEXT	[98,104-106]
Coping		
Coping Orientation to Problems Experienced Inventory (COPE-28)	CuidaTEXT	[98,104-106]
Affect		
Scale of Positive and Negative Experience (SPANE)	CuidaTEXT	[98,104-106]
Self-perception of negative and positive aspects of caregiving		
Carers of Older People in Europe (COPE) Index	InformCare	[102,103,107]
Positive aspects of caregiving		
Positive Aspects of Caregiving (PAC) Scale	FITT-C^b^	[44-50]
	CuidaTEXT	[98,104-106]
Self-efficacy		
PROMIS Self-Efficacy for Emotions (derived from the Self-Efficacy for Managing Chronic Disease (SEMCD) questionnaire)	Care Consultation or Care Consultation Plus	[41]
Self-Efficacy Questionnaire (SEQ)	FITT-C^b^	[44-50]
	FITT-D^e^	[43]
	RCTM^c^	[54-60]
Mastery		
Caregiving Mastery Index (CMI)	Laver^a^	[51-53]
Sense of capability		
Short Sense of Competence Scale (SSCQ)	Dementelcoach	[61-63,67,68,96]
	RCTM^c^	[54-60]
Caregiving competence		
Preparedness for Caregiving Scale (PCS)	CuidaTEXT	[98,104-106]
Psychological well-being		
World Health Organization Well-Being Index (WHO-5)	InformCare	[102,103,107]
Happiness		
TOPICS-MDS^g^ item	Dementelcoach	[61-63,67,68,96]
Quality of life		
Euro Quality of Life Visual Analog Scale (EQ-5D VAS)	FITT-C^b^	[44-50]
TOPICS-MDS^g^ item	Dementelcoach	[61-63,67,68,96]
Health		
PROMIS Global Health: Global Physical Health (GPH) and Global Mental Health (GMH)	Care Consultation or Care Consultation Plus	[41]
General Health Questionnaire (GHQ-28)	Dementelcoach	[61-63,67,68,96]
SF 36 General Health	FITT-D^e^	[43]
Not specified (1-item question)	CuidaTEXT	[98,104-106]
Unmet needs		
Measure of Unmet Needs (UN)	CuidaTEXT	[98,104-106]
Perceived social support or support for caring		
Multidimensional Scale of Perceived Social Support (MSPSS)	FITT-D^e^	[43]
	InformCare	[102,103,107]
Interpersonal Support Evaluation List (ISEL-12)	CuidaTEXT	[98,104-106]
Support for Caring subscale of the Adult Carer Quality of Life Questionnaire (AC-QoL)	RCTM^c^	[64,65]
Family functioning		
Family Assessment Device (FAD)	FITT-C^b^	[44-50]
	FITT-D^e^	[43]
Knowledge		
Alzheimer’s Disease Knowledge Test	FITT-D^e^	[43]
Epidemiology/Etiology Disease Scale (EEDS)	CuidaTEXT	[98,104-106]
Perceived change		
Perceived Change Scale (PCS)	Laver^a^	[51-53]
Secondary role strains		
Not specified (2 single-item ratings of the caregiver’s and the relative’s adjustment to residential long-term care placement)	RCTM^c^	[54-60]
Residential care stress		
Not specified (6-item measure for perceptions of staff communication with family, 5-item measure for staff support for family, 10-item measure assessing 5 positive and 5 negative types of caregiver interactions with their relative, staff, other family; item caregivers’ upset to see their relative in a residential care setting); Family Involvement Interview	RCTM^c^	[54-60]
Resource use		
Number of community or health services used	Coyne^a^ comparator or experimental	[42]
	FITT-C^b^	[44-50]
	FITT-D^e^	[43]
Outcomes for persons with dementia		
Depression		
Geriatric Depression Scale (GDS)	De Cola^a^	[82]
Global cognitive state		
Mini-Mental State Examination (MMSE)	De Cola^a^	[82]
Cognitive impairment		
Bedford Alzheimer Nursing Severity Scale (BANSS)	De Cola^a^	[82]
Functional dependency		
Activities of Daily Living (ADL) and Instrumental Activities of Daily Living Scale (IADL)	De Cola^a^	[82]
Instrumental activities of daily living		
Functional Activities Questionnaire (FAQ)	CuidaTEXT	[98,104-106]
Functionality		
Caregiver Assessment of Function and Upset (CAFU)	Laver^a^	[51-53]
Neuropsychiatric symptoms or behavioral symptom severity		
Neuropsychiatric Inventory (NPI) Total symptoms	Dementelcoach	[61-63,67,68,96]
Neuropsychiatric Inventory-Questionnaire (NPI-Q) Severity	Dementelcoach	[61-63,67,68,96]
	CuidaTEXT	[98,104-106]
Psychiatric symptoms		
Brief Psychiatric Rating Scale (BPRS)	De Cola^a^	[82]
Number of behaviors such as verbal aggression, refusing care, restlessness, anxiety, waking overnight, and repetitive questioning		
Caregiver Behavioral Occurrence and Upset Scale (modeled on the Agitated Behavior in Dementia Scale)	Laver^a^	[51-53]
Resource use		
Number of community or health services used	Coyne^a^ comparator or experimental	[42]
	FITT-C^b^	[44-50]

a When no name is reported, the name of the first author was assigned to the intervention.

b FITT-C: Family Intervention: Telephone Tracking–Caregiver.

c RCTM: Residential Care Transition Module.

d TeleFAMILIES: Telehealth-Administered Families Access to Memory Impairment and Loss Information, Engagement, and Supports.

e FITT-D: Family Intervention: Telephone Tracking–Dementia.

f ICSS: Internet-Based Caregiver Support Service.

g TOPICS-MDS: The Older Persons and Informal Caregivers Survey Minimum DataSet.

#### Results of Individual Sources of Evidence

We summarized the included interventions into groups that were formed based on the ICT used and the additional components of the interventions. We found 17 interventions that delivered counseling via telephone, email, or videoconferencing [41-50,66,70-78,83-87,93,94,108]. Three interventions provided counseling via videoconferencing [69,79,88]. One intervention each was assigned to the groups “counseling via email” [95], “counseling via SMS text messaging” [98,104-106], and “counseling via an interactive mobile app combined with additional features” [89]. We included 4 web-based psychosocial interventions that combined information, communication, and counseling [80,81,90-92,99-103,107]. Four videoconference- or telephone-based interventions combined counseling services with telemonitoring or psychoeducation [51-60,64,65,82,109]. Three technology-assisted interventions offered counseling as part of a comprehensive program with non-technology-assisted components [61-63,67,68,85-87,96,97].

The interventions aim to support family caregivers and persons with dementia, to provide information and education, and to enhance coping with and managing of the caregiving process. Theoretical foundations of interventions such as psychological or psychosocial concepts are referred to for 10 interventions [43-65,67,68,70,93-98,104-106,108]. The health service usage behavior has been theorized for an internet-based information support and personalized email intervention [90-92,99-101]. A program theory is mentioned for 1 intervention [83].

In the included reports, various components of the interventions are described, which are combined in different ways: intervention manuals or guidelines are made available for counselors. In some cases, special training is provided on dementia, counseling strategies, or the use of ICT in counseling. Supervision and coaching are offered to persons delivering counseling. There are various modes of documenting the content of the counseling sessions (eg, protocols and log sheets) and making it available to counselees (eg, letters and script proposals). In addition, information and educational material is offered as brochures, manuals, databases, websites, and videotapes. In some cases, the necessary technical equipment or assistance with its use is provided. Persons delivering counseling have qualifications in nursing, social work, social science, psychology, mental health, geriatrics, or occupational therapy. Additional experiences in gerontological, psychogeriatric, or dementia care, as well as experiences as family caregivers, are described.

Data on implementation issues is available to varying degrees. Implementation strategies were described for 2 interventions [55,96]. The reported modifications of interventions are mainly related to adaptations to the needs of new target groups and to pandemic-related restrictions. Barriers to implementation are technical issues (eg, lack of hardware, limited software functionality or usability, limited internet access, and suboptimal infrastructure), lack of digital skills (of persons delivering and receiving counseling), security issues, and aspects resulting from the type of technology used in each case (eg, loss of context, lack of prompts from the surroundings, and not being able to follow up). Facilitating factors are special trainings (eg, technical support and conversation strategies), ongoing support (eg, regular team meetings and supervision), features of interventions (eg, customized to sociocultural preferences of target groups and undemanding technology), and commitment of management of implementing institutions. External conditions of implementation are described as established organizations in which intervention programs are embedded, collaborations with interest-holders in dementia care, and the SARS-CoV-2 pandemic.

A detailed description of the included intervention programs is provided in Multimedia Appendix 3. Intervention components that have been incorporated into the program theory and that are represented in the logic model are referred to in the section Logic Model and Program Theory.

Outcomes of caregivers or persons with dementia examined and the assessment instruments used in the included studies are listed in Table 1.

### Qualitative Substudy

#### Sample Characteristics

We conducted 4 individual and 3 group interviews lasting from 1 to 3 hours (total duration: 12 hours 41 minutes) with 15 family caregivers (14 women and 1 man). One participant cared for a person with Parkinson disease; all other caregivers cared for a person with dementia. One family had a migration background; the caregiving arrangement no longer existed in 3 cases due to the death of the person receiving care.

In addition, 8 individual and 2 group interviews lasting from 50 to 100 minutes (total duration: 12 hours 14 minutes) with 12 counselors (11 women and 1 man) were conducted. Participants had been working as counselors for an average of 9 years and had qualifications in nursing, health and nursing science, social work, psychology, and administration. The providing institutions of participating counselors varied. Details on the characteristics of participants are provided in Table 2.

**Table 2.** Sociodemographic information on interview partners.

Characteristics	Values
Caregivers (n=15)	
Age in years, mean (range)	70 (44-83)
Gender, n	
Women	14
Man	1
Care-receiving person, n	
Husband or spouse	10
Mother	3
Father	1
Brother	1
Caregiving arrangement, n	
Home-based care	12
Institutionalized care	3
Years of caregiving, mean (range)	6 (1-20)
Counselors (n=12)	
Age in years, mean (range)	51 (33-61)
Gender, n	
Women	11
Man	1
Years of counseling, mean (range)	9 (1-30)
Providing organization, n	
Municipality	4
Welfare organization	2
Self-help organization	2
Research institution	1
University hospital	1
Registered association	1
Self-employed counselor	1

#### Themes Identified in Interviews With Caregivers

We identified the following 3 themes in the caregivers’ statements: being affected, feeling insecure and helpless in the face of the health care system, and search for information and communicative exchange.

##### Being Affected

*...and then I cried a lot and had really bad thoughts.*

[Caregiver 15]

Participants’ descriptions of how they are affected by the demands of the caregiving situation made up a large part of the interviews. The caregivers report conflicts with the persons receiving care encompassing harassment and insults, as well as verbal and physical attacks: “Back in November, my husband pushed me down the stairs” [Caregiver 15]. They also describe their grief over the loss of a person close to them due to the changes evoked by dementia, as well as the loss of life plans and perspectives. In addition, the demands of caregiving pose a burden on caregivers: “...that you just can’t do it anymore” [Caregiver 15].

##### Feeling Insecure and Helpless in the Face of the Health Care System

*And nobody tells you all this.*

[Caregiver 15]

The interviewees state a lack of information and a lack of support from persons or institutions involved in health care such as primary care physicians or health insurances:

*There’s the 24-hour care, every single day. Yeah, and then if you spend the whole morning on the phone just trying to get a simple answer, well, that’s just how it is for me.*

[Caregiver 15]

High financial expenses often limit the use of support services.

##### Search for Information and Communicative Exchange

*That made a huge difference for me, that I stopped thinking that I was somehow (...) weird.*

[Caregivers 3-5]

Caregivers report beneficial experiences with empathetic counseling focused on problem-solving:

*She [counselor] asks, “So, how are you doing?” Finally, someone actually asks how I’m handling all this. (...) And then she says, “Okay, so here’s what you can do...” – and that’s the kind of support that really does you good.*

[Caregivers 12-14]

Participants are not always aware of the differing objectives of services such as counseling, support, or self-help groups; however, they emphasize the importance of continuity in support services:

*It has to be done continuously. (...) Even though I was really impressed by my first two meetings here – and they were incredibly helpful – if I had stopped then, I wouldn’t have this feeling now.*

[Caregivers 3-5]

Interviewees indicate a preference for face-to-face meetings and express a reluctance to use ICT for counseling services: “You have to deal with all that tech stuff, and honestly, no – it’s just too much for me” [Caregiver 15]. Participants, however, make use of familiar technologies to maintain ongoing contact with their counselors: “I have her (counselor) on (messenging service), and she texts too – like she’ll wish me a nice weekend and ask if everything’s okay or if something’s going on” [Caregiver 15].

### Themes Identified in Interviews With Counselors

We identified the following themes in counselors’ interviews: work attitude and standards, unpredictability, expectations, working conditions, organizational influence, and tools: techniques and networking.

#### Work Attitude and Standards

*Good counseling is when you understand the person in their (...) lifeworld (...) when they feel that their issues are taken seriously.*

[Counselor 7]

The interviewees show a high level of commitment, appreciation, and empathy toward those seeking counseling. They describe how their work has fostered both personal and professional growth, and they highlight specific skills required for different counseling formats, such as anonymous helpline services. To further ensure the quality of counseling services, training and support offered to counselors are considered essential:

*So, as the head of the Support Center for Family Caregivers, I really keep an eye on that. What do my colleagues need? And they get it. It’s really important to me that the quality here is good.*

[Counselor 6]

To establish a common understanding of counseling, conceptual frameworks or manuals are developed to document the shared values: “I’m gonna write down what the core things in our counseling are – what’s really important to us” [Counselors 3 and 4]. Efforts are being made to reach out to family caregivers, for instance, by maintaining a presence in public spaces: “I’ve had this idea for a while – that we should have a place right in the middle of the city, like in a department store or a shopping center, somewhere people actually go” [Counselor 6]. This seems necessary, since family caregivers are often unable to initiate contact, according to the counselors:

*(Caregiver) says, “Yeah, your address has been sitting here for two years before I even call.” Happens all the time. All the time.*

[Counselor 6]

#### Unpredictability

*And that they [caregivers] come with such a mountain of problems.*

[Counselor 12]

Study participants indicate that the concerns and the state of mind of the caregivers are not known before the counseling and cover a wide spectrum—from “calm” to “nervous breakdown.” In some cases, counselees are overwhelmed and unable to speak:

*Sometimes they just sit there and start to cry. Then there’s something to drink, a tissue, and maybe even a piece of chocolate.*

[Counselor 6]

#### Expectations

*I wish they [caregivers] would open up so that they could really be helped properly.*

[Counselor 10]

We found that counselors have various expectations of those seeking counseling. While some of the participants stated that they had no expectations at all, others expected caregivers’ openness to share their concerns and experiences with the counselors. In some cases, counselors expect the caregivers to be willing to implement counselors’ advice.

#### Working Conditions

*Our doors are always open, and a colleague can come and say, “I have to tell you this.”*

[Counselor 6]

The description of the working conditions is a frequent theme in the interviews. The study participants highlight the importance of mutual support from colleagues as described in the headline quote. An important aspect is whether the participant is working in a team or as a “lone fighter”:

*And because there’s no real exchange with others, you have the chance to think about things and maybe do them differently – but you can never really share it with anyone.*

[Counselor 7]

Teams in which different professional groups are represented and whose members support each other are considered as an essential element in coping with the demands of counseling work:

*We’ve got people from all sorts of backgrounds – social work, sociology, (...) nursing background (...) psychologist, (...) gerontology and even theology. (...) we’re a really diverse team and can bring in all kinds of different perspectives.*

[Counselors 3 and 4]

The theme also covers personnel and technical equipment, which varies greatly among participants:

*I’d like to be able to scan some documents and bring them here to save people a trip. Maybe use a small mobile printer or something like that (...) but there’s just no way.*

[Counselor 9]

In addition, the opportunity to independently organize and design their everyday working life is considered a crucial factor of the working conditions.

#### Organizational Influence

*Then they [superiors] would clearly say, “That’s not your job!”*

[Counselor 7]

The providing organization has a significant influence on counselors’ performance in the sense of a spillover effect. Lack of agreement between managers and counselors on the purpose and the design of counseling services has a negative impact on its implementation.

#### Tools: Techniques and Networking

*To pave the way for people.*

[Counselor 9]

Study participants describe techniques they are using in their professional lives. These include conversation management, individual practices to ease the situation for counselees (eg, offering something to drink and asking questions that facilitate the dialogue), as well as assessment tools, documentation aids, and information materials. ICT is used to enhance access to counseling services:

*If people live further away, that’s totally normal. Or if they’re working, they’ll say, “Okay, I can chat with you for fifteen minutes on (videoconferencing software).” We’re happy to do that.*

[Counselor 6]

Professional networks are used to help caregivers with problems that fall outside the counselors’ scope of responsibility (eg, medical and legal issues). Cooperation (rather than competition) between different service providers and the combination of various support services is seen as key to providing effective support:

*Well, just counseling on its own or just training doesn’t really work. It has to be a combination of different things – then it can work.*

[Counselor 8]

### Constitutive Pattern

The common ground in the lifeworld perceptions of the two interest-holder groups forms the constitutive pattern of *somebody to count on*. While caregivers express the need for *having somebody to count on*, who guides them empathically through the challenges of family dementia care, counselors aim at *being somebody to count on* by competently assisting caregivers to master those challenges.

### Outcomes Identified by Interview Partners

Caregivers and counselors described effects, which could be used to measure effectiveness of counseling. The potential outcomes are listed in Table 3.

**Table 3.** Outcomes identified by interview partners (caregivers or counselors).

Outcomes	Identifying interview partner
Level of knowledge	Ca^a^; Co^b^
Knowledge about the disease and its (potential) course	Ca; Co
Knowledge about how to deal with changed behavior of the person with dementia	Ca; Co
Knowledge about what to do to improve well-being and safety of the person with dementia	Ca; Co
Knowledge about available support services	Ca; Co
Knowledge about legal regulations and financial support options	Co
Level of caregiver burden	Co
Level of caregiver distress	Co
Level of emotional strain	Co
Level of emotional overload	Co
Stability of caregiver perceived burden (level is maintained despite increasing care needs)	Co
Sense of relief	Ca; Co
Sense of relief through empathetic understanding	Ca; Co
Sense of relief from having a counselor to fall back on (reliability, accountability of counseling)	Ca; Co
Sense of relief through active support (eg, support in filling out application forms)	Ca; Co
Sense of order (plan in mind about what to do next or how to get along with caregiving)	Ca; Co
Caregiver depressive symptoms	Ca
Stability of caregiver perceived depressive symptoms (level is maintained despite increasing care needs)	Co
Caregiver guilt	Co
Feeling of helplessness	Ca
Quality of life (caregiver)	Co
Quality of life (person with dementia)	Co
Caregiver well-being	Co
Caregiver (physical or psychological) health	Co
Caregiver satisfaction	Co
Congruousness of caregiving arrangement with caregiver’s preferences and wishes	Co
Caregiver attitude toward caregiving	Co
Sense of confidence	Co
Sense of confidence in decision-making	Co
Sense of confidence in managing caregiving challenges	Co
Caregiver mastery	Co
Empowerment (eg, in finding support by oneself)	Co
Feeling well advised	Co
Problem-solving ability	Ca; Co
Realization of counselors’ suggestions (eg, changes in the home environment, use of support services, creation or use of personal space, and hobbies)	Co
Balance between caregiving and self-care	Co
Recognition of personal limits in caregiving capacity	Co
Safety of care	Co
Stability of caregiving arrangement over time	Co
Number of support services used	Co
Duration of caregiving arrangement	Co
Time to transition to long-term care facility	Co
Extent and stability of caregiver’s support network	Co
Number of conflicts with care-receiving person	Co
Cost of care	Co

a Ca: caregivers.

b Co: counselors.

Taken together, the statements of the two participant groups on effective counseling include the following characteristics: an empathetic, appreciative, and compassionate attitude; knowledge transfer; provision of materials and help with bureaucracy; providing a view from the “outside”: classifying, weighting, and sorting caregiving issues; recommendations for action (eg, dealing with changes in behavior); support in decision-making and in defining boundaries; and sustainability and continuity. Comments and suggestions of participating caregivers and counselors that have been incorporated into the program theory and that are represented in the logic model are referred to in the section Logic Model and Program Theory.

### Logic Model and Program Theory

**Figure 2.**
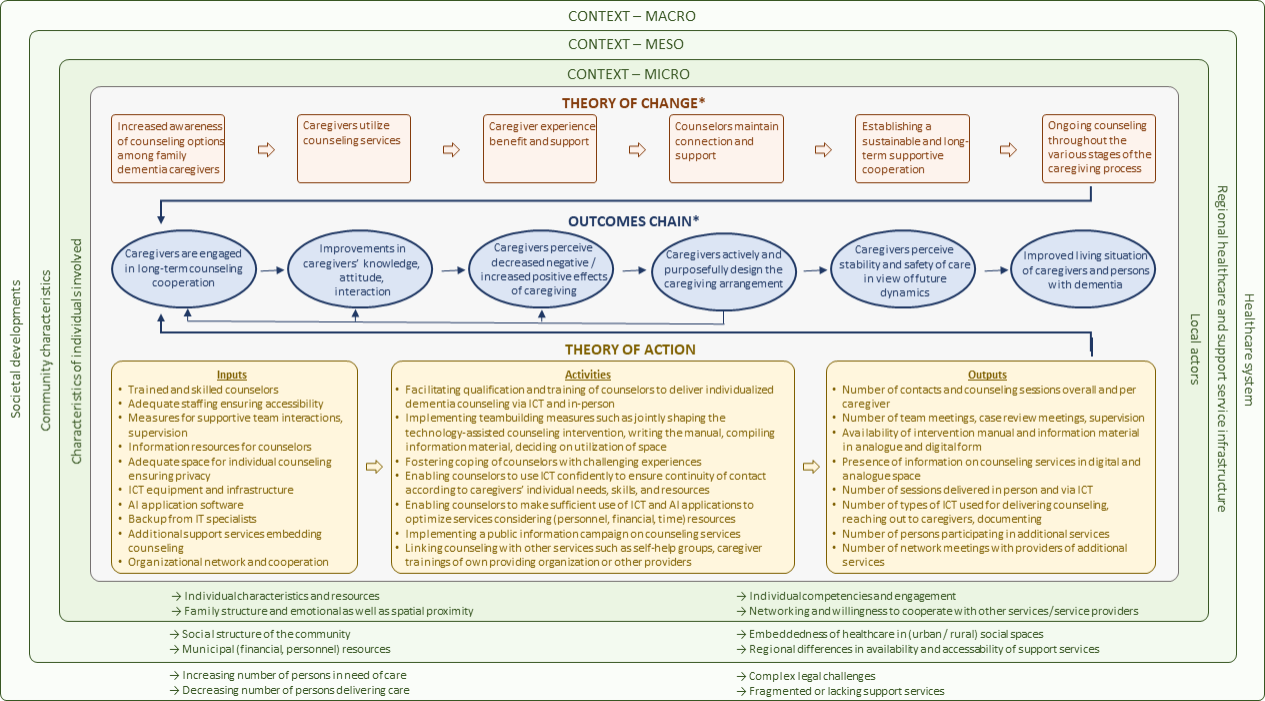
Logic model of the technology-assisted counseling intervention. AI: artificial intelligence; ICT: information and communication technology. *The theory of change, the outcomes chain, and the outcomes listed in Tables1  3 will be subject to a future consensus-building process.

The logic model of the technology-assisted counseling intervention is provided in Figure 2.

In the following section, we outline the program theory. The detailed version can be obtained from the authors on request.

The contextual factors of family dementia care in Germany on the macro-, meso-, and microlevels are illustrated in green in the logic model: Ongoing demographic developments lead to an imbalance between care needs and families’ capacity to provide care. Challenges faced by individuals assuming caregiving responsibilities can be exacerbated by fragmented health care services and complex legal regulations. The unequal distribution of support services across communities with varying resources can result in inadequate support for family caregivers of persons with dementia.

The problem to be addressed is the inadequate use of counseling services by family caregivers of persons with dementia. Study participants indicated that caregivers are rarely made aware of the counseling services available. Moreover, caregivers often do not actively seek contact with services because they feel overwhelmed by caregiving tasks, experience feelings of shame, or hesitate due to the perceived effort involved.

In our theory of change, we outline how a technology-assisted counseling intervention may help to address this issue: We hypothesize that an increased awareness of counseling services among family dementia caregivers leads to an initial use associated with a beneficial and supportive experience.

As both caregivers and counselors emphasize the importance of continuous counseling and support, we propose that counselors adopt unobtrusive strategies to maintain engagement with caregivers. Using diverse types of ICT that caregivers are familiar with facilitates the ongoing contact with counselors. This approach fosters a sustainable, long-term supportive cooperation that enables consistent counseling throughout all stages of the caregiving process.

In the outcomes chain, we describe the immediate and intermediate outcomes, which were derived by clustering outcomes identified in the literature and those mentioned by study participants (refer to Tables 1 and 3). We assume that long-term counseling cooperation leads to improved caregiver knowledge about caregiving aspects (eg, the (potential) course of dementia, how to deal with changed behavior of the person with dementia, and available support services), to an enhanced attitude toward caregiving, and to improved interaction with the care-receiving person. This entails an altered impact of caregiving, in that the caregivers perceive decreased negative effects such as burden, distress, feeling of helplessness, and increased positive effects such as sense of confidence, empowerment, and satisfaction. Caregivers are thereby enabled to actively and purposefully shape the caregiving arrangement, for example, by using support services and to achieve congruousness of the caregiving arrangement with their own preferences and values. Caregivers perceive stability of the caregiving arrangement and safety of care despite unpredictable developments and dynamics and are aware that they may approach counselors any time needed. This leads to an improved living situation for both the caregivers and the persons with dementia. As indicated by the arrows in the logic model, the levels of the outcomes chain can occur in a recursive process.

Within the theory of action, we specify the inputs and actions through which the theory of change can be initiated [17]. The inputs specified in the theory of action are derived from the statements of study participants presented above and from intervention components described in the literature (refer to Multimedia Appendix 3). As counselors emphasized the importance of adequate working conditions, we incorporated aspects such as sufficient staffing, measures to promote a supportive working environment, and access to informational resources, including libraries and databases. Appropriate technological equipment and infrastructure, along with IT support, enable the reliable delivery of technology-assisted counseling. Furthermore, networks for cooperation with complementary services—either within the organization or in collaboration with external providers—are crucial for providing effective support. Program activities are directed at enabling counselors to provide technology-assisted counseling to family dementia caregivers competently and confidently. The activities depicted in the logic model further promote supportive working relationships by fostering mutual support within the team and articulating shared values in their work, for example, through manuals or counseling concepts. The implementation of a public information campaign may help to raise awareness of counseling services and facilitate outreach to family caregivers. In addition, diverse services are integrated to ensure timely, individualized support. Considering limited personnel, financial, and time resources, counselors are empowered to use ICT and artificial intelligence (AI) applications sufficiently to optimize the efficiency of services. Outputs represent the tangible, measurable products of activities [17]. We included the availability of the intervention manual and the information material, as well as the number of persons using counseling or other services and the number of events such as meetings to enhance cooperation within the team, within the organization, and with other organizations, as well as counts of types of ICT.

## Discussion

### Principal Findings

In this paper, we present the results of a scoping review and a qualitative study synthesized into a logic model and a program theory of a technology-assisted counseling intervention for family caregivers of persons with dementia. Information obtained from literature, empirical findings from the qualitative substudy, and theoretical approaches have been merged to guide the future COS consensus process and the modeling of the intervention.

### Theoretical Approaches and Implications of Evolving Technological Modalities Informing the Program Theory

Presumed mechanisms of the technology-assisted counseling intervention were informed by theoretical approaches underpinning the interventions identified in the scoping review: The model of determinants of subjective burden of caregivers of persons with dementia forms the theoretical framework of the Dementelcoach intervention [68]. Aspects such as caregivers’ personal characteristics, material and social circumstances, and the support they receive are integrated into the program theory. In addition, the concept map of the Internet-Based Caregiver Support Service (ICSS) described by Chiu and Eysenbach [92] to conceptualize usage behavior of family caregivers by integrating 3 theoretical approaches (Anderson’s model of health service utilization, Venkatesh’s theory of technology acceptance, and Chatman’s and Wilson’s information behavior theories) was used to shape our theoretical understanding. Factors such as dynamic and individual caregiver needs, perceived efforts of ICT options, and preferences in using ICT [92] have been taken into account.

Further findings of the scoping review illustrate how technologies used for delivering counseling have evolved over time from technology-assisted counseling provided exclusively via telephone to counseling via chats or SMS text messaging. In addition, multiple technologies are used in some interventions for establishing contact or delivering counseling. This diversification has led to a partial overlap in the categories formulated to group interventions in this paper.

The appropriateness of technological options for administering counseling varies with respect to availability, flexibility, and requirements for technological equipment and skills [3,8]. In addition, the suitability of the various technological approaches for discussing personal issues in depth or addressing specific requests directly is perceived differently [3,8]. Therefore, we will integrate multiple technological ways to access counseling in the development of the intervention to accommodate caregivers’ individual needs and resources and to enhance technology acceptance.

The combination of various ICT systems poses higher demands on counselors’ technological literacy and counseling competencies. To effectively switch between various modes of delivery, counselors require specific qualifications and skills, for example, to compensate for the lack of visual clues during telephone counseling, to facilitate openness in conversations via videoconferencing software, or to address counselees’ concerns in written asynchronous communication such as email or chat [8]. To enable counselors to use ICT confidently, inputs of the intervention aim at promoting an effective working environment by including adequate technological equipment and infrastructure as well as available support by IT specialists for training counselors and troubleshooting.

### Overcoming Utilization Barriers Through Integrated Access Modalities and Targeted Outreach

Caregivers and counselors participating in the qualitative study largely expressed a preference for in-person counseling. The reluctance to use ICT that we observed despite the shift toward technology-assisted services during the SARS-CoV-2 pandemic [110] could have been partly influenced by the older age of the participating caregivers. Nevertheless, Gonzalez-Fraile et al [3] state in their systematic review that remotely delivered training and support interventions appeared to be less acceptable than control interventions, as assessed by attrition rates. Therefore, we integrated face-to-face services offered at counseling centers or in caregivers’ home environments into the program theory of the technology-assisted counseling intervention.

We hypothesize that the combination of diverse technologies in addition to in-person counseling contributes to overcoming barriers to utilization of counseling services. The different characteristics of technological options allow for the targeting of distinct user groups. Findings of the qualitative study indicate that SMS text messaging is rather highly accepted among middle-aged or older persons and can be used to maintain contact and emotional involvement by sharing photos or invitations to events. Results from the literature also show that SMS text messaging is suitable for addressing disparities in access to caregiving support for persons belonging to minorities [98,106]. The needs of ethnic groups may also be met by counseling via email, offering an alternative service model [99,100]. Participants of the qualitative substudy stated that nonnative speaking persons may benefit from conversations via email as the asynchronous communication gives them more time to consider counselors’ suggestions.

As outlined above, caregivers described in detail the consequences arising from the caregiving responsibility summarized in the theme “being affected.” Feeling overwhelmed and stressed can prevent caregivers from actively seeking information and support [111]. In addition, the lack of awareness as well as the regional lack of availability contributes to the underuse of support services such as counseling [111]. We therefore included a campaign in the program theory to increase awareness among public and professional communities about counseling opportunities. By reaching out through an enhanced digital and analog presence, we aim to encourage family dementia caregivers to initiate contact to counseling services.

### Embedding Counseling Services for Maintaining Ongoing Contact

Inquiries about the caregivers’ mental models during the interviews indicate that family dementia caregivers often do not distinguish between the different objectives of various support services. While professionals intend to give room for sharing caregiving experiences in support groups, for example, some of the participants also expected information to be conveyed at these meetings. Embedding counseling in a variety of support services may contribute to supplementing the supportive effect of individual interventions, to maintaining contact, and to actively offering support when need occurs. Interventions offering counseling as part of a comprehensive program with non-technology-assisted components provide examples of such an approach: One example combines telephone counseling with a group activity program for community-dwelling individuals with dementia, aiming to empower persons with dementia and their caregivers [97]. Another combines telephone coaching (Dementelcoach) with respite care, thereby enhancing the effectiveness of the intervention [68]. A third example links two eHealth caregiver interventions to existing Meeting Centers for persons with dementia and their family caregivers [63,96].

### Integrating AI Technology to Mitigate the Shortage of Skilled Health Care Professionals

With care needs increasing due to the demographic development, the shortage of skilled health care workers results in higher workloads and accelerated turnover rates [112]. Various strategies are described for retaining health care staff [112]. Authors of the Dementelcoach study reported that professionals delivering the intervention were part-time workers in the psychogeriatric sector. These professionals were encouraged to expand their work hours to provide counseling in conjunction with their existing jobs [68]. Offering a complementary activity may help to meet existing support needs in the face of the shortage of skilled staff [68].

All participating counselors reported a high workload, and the growing scarcity of skilled professionals may further exacerbate work-related stress [113]. To reduce time spent on documentation and administrative tasks, AI technology will be integrated into the intervention at the level of partial automation by augmenting human performance [113]. In using large language models for transcribing counseling sessions and composing summaries, counselors are relieved of documentation tasks and allowed to focus solely on counselees’ concerns during sessions. AI can also be used for scheduling tasks and to compile lists of regionally available support services for caregivers based on an AI-augmented database [114]. AI-drafted content will be evaluated by counselors to ensure accuracy and quality of information. This may help to alleviate concerns associated with the use of AI in the context of vulnerable groups—in particular, concerns about the reliability of information, privacy, and data security [113].

In sum, technology can make an important contribution to overcoming problems arising from the increasing need for support due to the growing number of persons with dementia [6], the growing shortage of skilled health care professionals [115], and social and infrastructural inequalities [116]. It is essential that technology-assisted counseling interventions are designed with consideration of individual and situational requirements in order to enhance their acceptability and feasibility from the perspectives of both recipients and providers [117].

### Strengths and Limitations

We established a profound database for formulating an initial program theory and preparing for the development of a COS by integrating knowledge obtained from literature and the lived experience of two interest-holder groups. Participants in the qualitative study were recruited all over Germany. Caregivers living in urban and rural regions shared their experiences with counseling services, but we did not succeed in recruiting a heterogeneous sample in terms of age and gender. This may impose limitations on findings, as perspectives of younger and diverse caregivers, who might be more technologically savvy, are underrepresented. Younger family caregivers who are also employed, particularly long-distance caregivers, may be more inclined to use ICT to access counseling services. By combining various technological access options with in-person counseling, we advocate for an inclusive approach that addresses the needs of diverse target groups.

We were able to identify commonalities and differences in the interest-holders’ expectations and mental models, which were incorporated into the program theory.

Another limitation is the inclusion of only two groups of interest-holders. Due to the limited duration of the project, we were not able to expand the study population of the qualitative inquiry. Representatives of providing organizations and policymakers will be included in the consensus process of the COS.

We followed accepted methodological guidance [13,17] and integrated methodological approaches in an innovative way. This proved to be beneficial: When asked about potential outcomes for measuring effectiveness of counseling, interviewees reflected on and critically questioned their own ideas and expectations. We found it advantageous to consider adequate outcomes from the beginning of the development process, to debate underlying (hypothesized) causal relationships, and to visualize causal assumptions in the logic model. This theory-led approach to the development, implementation, and evaluation of a technology-assisted counseling intervention for family caregivers of persons with dementia is consistent with the recommendations of the updated UK Medical Research Council guidance [9].

### Future Directions

We will perform a theory-led approach to modeling, implementing, and evaluating the technology-assisted counseling intervention for family dementia caregivers. In a first step, the “long list” of outcomes will be created in accordance with the COS development methodology [13]. The participants of the qualitative study found it surprisingly difficult to name potential outcomes for assessing the effectiveness of counseling interventions, which led to a high number of (partially) overlapping outcomes. In a follow-up study, we will involve representatives of interest-holder groups such as family caregivers, counselors, managers of provider organizations, and researchers to integrate outcomes reported in literature (Table 1) and outcomes identified by interview partners (Table 3): Redundant outcomes will be removed; for example, outcomes such as “burden,” “guilt,” and “stress or distress” were identified in both the literature and interviews. The remaining outcomes will then be grouped into outcome domains applying ontologies, as proposed by Williamson et al [13]. The resulting “long list” of clustered outcomes forms the basis for the consensus process of the COS [13]. We will apply the Delphi approach involving groups of relevant interest-holders to determine important clinical outcomes [13]. To establish a shared understanding among participating interest-holders, elements of the program theory will be gradually integrated into the consensus process. At the outset of this process, we will use the logic model to visualize and critically discuss the theory of change. Subsequently, the importance of individual outcomes will be rated in Delphi rounds and finally consented in a consensus conference [13].

The refined logic model and program theory will then be used for modeling the intervention in collaboration with practice partners in a future research project. The logic model will serve to foster a shared understanding among the individuals involved and to systematically document the adaptation of key components to the specific context [9,18].

### Conclusions

We developed an initial program theory of a technology-assisted counseling intervention for family caregivers of persons with dementia by introducing a methodological innovation. Findings obtained from interest-holder groups and literature are synthesized into a program theory and visualized by a logic model. We have also compiled a comprehensive list of potential outcomes, which includes the outcomes examined in clinical studies and those that are relevant from the perspective of interest-holders. This enables the consensus process for finalizing the COS for technology-assisted counseling interventions.

These results will inform the theory-led modeling, implementation, and evaluation of the intervention, which will include a customized ICT package. This package has the potential to improve accessibility to counseling for caregivers by overcoming disparities in access to health care services. In addition, the design of the intervention can positively impact work conditions for health care professionals delivering support and improve the efficiency of services.

## Supplementary material

10.2196/81669 Multimedia Appendix 1 Search strategies.

10.2196/81669 Multimedia Appendix 2 Interview guide.

10.2196/81669 Multimedia Appendix 3 Description of reports and interventions.

10.2196/81669 Checklist 1 PRISMA-ScR, SRQR, and COS-STAR checklists.

## Abbreviations

AI: artificial intelligence
COMET: Core Outcome Measures in Effectiveness Trials Initiative
COS: core outcome set
COS-STAR: Core Outcome Set—Standards for Reporting
CReDECI 2: Revised Criteria for Reporting the Development and Evaluation of Complex Interventions
ICSS: Internet-Based Caregiver Support Service
ICT: information and communication technologies
PRISMA-ScR: Preferred Reporting Items for Systematic Reviews and Meta-Analyses, Extension for Scoping Reviews
ProCOS: Program Theory and Preparation of a Core Outcome Set Project
RCT: randomized controlled trial
SRQR: Standards for Reporting Qualitative Research
TIDieR: Template for Intervention Description and Replication
