# Supplementary material for: Program Theory and Core Outcome Set Development for a Technology-Assisted Counseling Intervention in Dementia: Multimethods Study
Source: J Med Internet Res. 2026 Jan 20;28:e81669. doi: 10.2196/81669 (PMC12818504; doi:10.2196/81669)
Supplement: Multimedia Appendix 1 [file jmir-v28-e81669-s001.docx]

**Database specific search strategies**

| **MEDLINE via PubMed**  Date: December 06, 2023  Filter: 2021-2024 | | |
| --- | --- | --- |
| **#** | **Entry** | **Hits** |
| 1 | ("dement*"[Title/Abstract] OR "alzheimer*"[Title/Abstract] OR "major neurocognitive disorder*"[Title/Abstract] OR "DEMENTIA"[MeSH Terms]) AND (2021:2024[pdat]) | 64,430 |
| 2 | ("APP"[Title/Abstract] OR "APPS"[Title/Abstract] OR "augmented realit*"[Title/Abstract] OR "BLENDED TREATMENT"[Title/Abstract] OR "BLENDED THERAPY"[Title/Abstract] OR "BLOG"[Title/Abstract] OR "cell hone*"[Title/Abstract] OR "cellphone*"[Title/Abstract] OR "CHAT"[Title/Abstract] OR "CHATS"[Title/Abstract] OR "chatbot*"[Title/Abstract] OR "computer*"[Title/Abstract] OR "digital*"[Title/Abstract] OR "E COUNSELING"[Title/Abstract] OR "ECOUNSELING"[Title/Abstract] OR "E COUNSELING"[Title/Abstract] OR "E COUNSELLING"[Title/Abstract] OR "ECOUNSELLING"[Title/Abstract] OR "E COUNSELLING"[Title/Abstract] OR "E HEALTH"[Title/Abstract] OR "EHEALTH"[Title/Abstract] OR "E HEALTH"[Title/Abstract] OR "electronic*"[Title/Abstract] OR "email*"[Title/Abstract] OR "e mail*"[Title/Abstract] OR "FACEBOOK"[Title/Abstract] OR "ICT"[Title/Abstract] OR "INTERNET"[Title/Abstract] OR "MESSENGER"[Title/Abstract] OR "MHEALTH"[Title/Abstract] OR "MHEALTH"[Title/Abstract] OR "MOBILE DEVICE"[Title/Abstract] OR "MOBILE HEALTH"[Title/Abstract] OR "MOBILE PHONE"[Title/Abstract] OR "MOBILE HEALTH"[Title/Abstract] OR "online*"[Title/Abstract] OR "PHONE"[Title/Abstract] OR "REMOTE"[Title/Abstract] OR "SKYPE"[Title/Abstract] OR "smartphone*"[Title/Abstract] OR "SMS"[Title/Abstract] OR "SOCIAL MEDIA"[Title/Abstract] OR "TABLET"[Title/Abstract] OR "TABLETS"[Title/Abstract] OR "technolog*"[Title/Abstract] OR "TELE HOME CARE"[Title/Abstract] OR "TELE MEDICINE"[Title/Abstract] OR "TELECARE"[Title/Abstract] OR "TELE-CARE"[Title/Abstract] OR "TELECARING"[Title/Abstract] OR "TELECARING"[Title/Abstract] OR "TELECOMMUNICATION"[Title/Abstract] OR "teleconference*"[Title/Abstract] OR "tele conference*"[Title/Abstract] OR "teleconsultation*"[Title/Abstract] OR "tele consultation*"[Title/Abstract] OR "TELEHEALTH"[Title/Abstract] OR "TELE-HEALTH"[Title/Abstract] OR "TELEMEDICINE"[Title/Abstract] OR "TELENURSE"[Title/Abstract] OR "TELENURSING"[Title/Abstract] OR "TELEPHONE"[Title/Abstract] OR "TELEPHONES"[Title/Abstract] OR "TELEPRESENCE"[Title/Abstract] OR "TELEPRESENT"[Title/Abstract] OR "TELEREHABILITATION"[Title/Abstract] OR "TELEREHABILITATION"[Title/Abstract] OR "teletherap*"[Title/Abstract] OR "tele therap*"[Title/Abstract] OR "TELETREATMENT"[Title/Abstract] OR "TELETREATMENT"[Title/Abstract] OR "VIDEOCONFERENCE"[Title/Abstract] OR "VIDEOCONFERENCES"[Title/Abstract] OR "VIRTUAL"[Title/Abstract] OR "WEB BASED"[Title/Abstract] OR "WEBBASED"[Title/Abstract] OR "WEB BASED"[Title/Abstract] OR "WORLD WIDE WEB"[Title/Abstract] OR "TELEMEDICINE"[MeSH Terms] OR "TELENURSING"[MeSH Terms] OR "TECHNOLOGY"[MeSH Terms] OR "VIRTUAL REALITY"[MeSH Terms] OR "TELECOMMUNICATIONS"[MeSH Terms] OR "VIDEOCONFERENCING"[MeSH Terms] OR "TELEREHABILITATION"[MeSH Terms] OR "INTERNET-BASED INTERVENTION"[MeSH Terms] OR "SOCIAL MEDIA"[MeSH Terms] OR "INFORMATION TECHNOLOGY"[MeSH Terms] OR "REMOTE CONSULTATION"[MeSH Terms] OR "DISTANCE COUNSELING"[MeSH Terms] OR "VIRTUAL REALITY"[MeSH Terms] OR "TELEPHONE"[MeSH Terms] OR "CELL PHONE"[MeSH Terms]) AND (2021:2024[pdat]) | 630,548 |
| 3 | ("advice*"[Title/Abstract] OR "advise*"[Title/Abstract] OR "ADVISING"[Title/Abstract] OR "coach*"[Title/Abstract] OR "consult*"[Title/Abstract] OR "COUNSELLING"[Title/Abstract] OR "COUNSELING"[Title/Abstract] OR "instruct*"[Title/Abstract] OR "guidance*"[Title/Abstract] OR "guide*"[Title/Abstract] OR "MENTORING"[Title/Abstract] OR "PASTORAL CARE"[Title/Abstract] OR "social support*"[Title/Abstract] OR "supervis*"[Title/Abstract] OR "support service*"[Title/Abstract] OR "talking therap*"[Title/Abstract] OR "MENTORING"[MeSH Terms] OR "COUNSELING"[MeSH Terms] OR "SOCIAL SUPPORT"[MeSH Terms]) AND (2021:2024[pdat]) | 409,784 |
| 4 | 1 AND 2 AND 3 | 1,188 |
| 5 | ("surgical"[Title/Abstract] OR "surgery"[Title/Abstract] OR "geneti*"[Title/Abstract] OR "proteomic*"[Title/Abstract] OR "genomic*"[Title/Abstract] OR "protein*"[Title/Abstract] OR "neuroimag*"[Title/Abstract]) AND (2021:2024[pdat]) | 1,079,303 |
| 6 | 4 NOT 5 | 1,036 |

| **CINAHL**  Date: December 06, 2023  Filter: 2021-2024 | | |
| --- | --- | --- |
| **#** | **Entry** | **Hits** |
| 1 | TI DEMENT* OR TI ALZHEIMER* OR TI "MAJOR NEUROCOGNITIVE DISORDER*" OR AB DEMENT* OR AB ALZHEIMER* OR AB "MAJOR NEUROCOGNITIVE DISORDER*" OR MH DEMENTIA+ | 18,964 |
| 2 | TI APP OR TI APPS OR TI "AUGMENTED REALIT*" OR TI "BLENDED TREATMENT" OR TI "BLENDED THERAPY" OR TI BLOG OR TI "CELL PHONE*" OR TI CELLPHONE* OR TI CHAT OR TI CHATS OR TI CHATBOT* OR TI COMPUTER* OR TI DIGITAL* OR TI "E COUNSELING" OR TI ECOUNSELING OR TI ECOUNSELING OR TI "E COUNSELLING" OR TI ECOUNSELLING OR TI E-COUNSELLING OR TI "E HEALTH" OR TI EHEALTH OR TI E-HEALTH OR TI ELECTRONIC* OR TI EMAIL* OR TI E-MAIL* OR TI FACEBOOK OR TI ICT OR TI INTERNET OR TI MESSENGER OR TI MHEALTH OR TI M-HEALTH OR TI "MOBILE DEVICE" OR TI "MOBILE HEALTH" OR TI "MOBILE PHONE" OR TI "MOBILE-HEALTH" OR TI ONLINE* OR TI PHONE OR TI REMOTE OR TI SKYPE OR TI SMARTPHONE* OR TI SMS OR TI "SOCIAL MEDIA" OR TI TABLET OR TI TABLETS OR TI TECHNOLOG* OR TI "TELE HOME CARE" OR TI "TELE MEDICINE" OR TI TELECARE OR TI TELE-CARE OR TI TELECARING OR TI TELE-CARING OR TI TELECOMMUNICATION OR TI TELECONFERENCE* OR TI TELE-CONFERENCE* OR TI TELECONSULTATION* OR TI TELE-CONSULTATION* OR TI TELEHEALTH OR TI TELE-HEALTH OR TI TELEMEDICINE OR TI TELENURSE OR TI TELENURSING OR TI TELEPHONE OR TI TELEPHONES OR TI TELEPRESENCE OR TI TELEPRESENT OR TI TELEREHABILITATION OR TI TELE- REHABILITATION OR TI TELETHERAP* OR TI TELE-THERAP* OR TI TELETREATMENT OR TI TELE-TREATMENT OR TI VIDEOCONFERENCE OR TI VIDEOCONFERENCES OR TI VIRTUAL OR TI "WEB BASED" OR TI WEBBASED OR TI WEB-BASED OR TI "WORLD WIDE WEB" OR AB APP OR AB APPS OR AB "AUGMENTED REALIT*" OR AB "BLENDED TREATMENT" OR AB "BLENDED THERAPY" OR AB BLOG OR AB "CELL PHONE*" OR AB CELLPHONE* OR AB CHAT OR AB CHATS OR AB CHATBOT* OR AB COMPUTER* OR AB DIGITAL* OR AB "E COUNSELING" OR AB ECOUNSELING OR AB E-COUNSELING OR AB "E COUNSELLING" OR AB ECOUNSELLING OR AB E-COUNSELLING OR AB "E HEALTH" OR AB EHEALTH OR AB E-HEALTH OR AB ELECTRONIC* OR AB EMAIL* OR AB E-MAIL* OR AB FACEBOOK OR AB ICT OR AB INTERNET OR AB MESSENGER OR AB MHEALTH OR AB M-HEALTH OR AB "MOBILE DEVICE" OR AB "MOBILE HEALTH" OR AB "MOBILE PHONE" OR AB "MOBILE- HEALTH" OR AB ONLINE* OR AB PHONE OR AB REMOTE OR AB SKYPE OR AB SMARTPHONE* OR AB SMS OR AB "SOCIAL MEDIA" OR AB TABLET OR AB TABLETS OR AB TECHNOLOG* OR AB "TELE HOME CARE" OR AB "TELE MEDICINE" OR AB TELECARE OR AB TELE-CARE OR AB TELECARING OR AB TELE-CARING OR AB TELECOMMUNICATION OR AB TELECONFERENCE* OR AB TELE-CONFERENCE* OR AB TELECONSULTATION* OR AB TELE-CONSULTATION* OR AB TELEHEALTH OR AB TELE-HEALTH OR AB TELEMEDICINE OR AB TELENURSE OR AB TELENURSING OR AB TELEPHONE OR AB TELEPHONES OR AB TELEPRESENCE OR AB TELEPRESENT OR AB TELEREHABILITATION OR AB TELEREHABILITATION OR AB TELETHERAP* OR AB TELE-THERAP* OR AB TELETREATMENT OR AB TELETREATMENT OR AB VIDEOCONFERENCE OR AB VIDEOCONFERENCES OR AB VIRTUAL OR AB "WEB BASED" OR AB WEBBASED OR AB WEB-BASED OR AB "WORLD WIDE WEB" OR MH TELEMEDICINE+ OR MH TELENURSING+ OR MH TECHNOLOGY+ OR MH VIRTUAL REALITY+ OR MH TELECOMMUNICATIONS+ OR MH VIDEOCONFERENCING+ OR MH TELEREHABILITATION+ OR MH INTERNET-BASED INTERVENTION+ OR MH SOCIAL MEDIA+ OR MH INFORMATION TECHNOLOGY+ OR MH REMOTE CONSULTATION+ OR MH TELEPHONE+ OR MH CELL PHONE+ | 161,675 |
| 3 | TI ADVICE* OR TI ADVISE* OR TI ADVISING OR TI COACH* OR TI CONSULT* OR TI COUNSELLING OR TI COUNSELING OR TI INSTRUCT* OR TI GUIDANCE* OR TI GUIDE* OR TI MENTORING OR TI "PASTORAL CARE" OR TI "SOCIAL SUPPORT*" OR TI SUPERVIS* OR TI "SUPPORT SERVICE*" OR TI "TALKING THERAP*" OR AB ADVICE* OR AB ADVISE* OR AB ADVISING OR AB COACH* OR AB CONSULT* OR AB COUNSELLING OR AB COUNSELING OR AB INSTRUCT* OR AB GUIDANCE* OR AB GUIDE* OR AB MENTORING OR AB "PASTORAL CARE" OR AB "SOCIAL SUPPORT*" OR AB SUPERVIS* OR AB "SUPPORT SERVICE*" OR AB "TALKING THERAP*" OR MH COUNSELING+ OR MH PSYCHOSOCIAL SUPPORT+ | 128,370 |
| 4 | 1 AND 2 AND 3 | 474 |
| 5 | TI SURGICAL OR TI SURGERY OR TI GENETI* OR TI PROTEOMIC* OR TI GENOMIC* OR TI PROTEIN* OR TI NEUROIMAG* OR AB SURGICAL OR AB SURGERY OR AB GENETI* OR AB PROTEOMIC* OR AB GENOMIC* OR AB PROTEIN* OR AB NEUROIMAG* | 146,281 |
| 6 | 4 NOT 5 | 447 |

| **CENTRAL via Cochrane Library**  Date: December 08, 2023  Filter: publication date between Jan 2021 and Dec 2023 | | |
| --- | --- | --- |
| **#** | **Entry** | **Hits** |
| 1 | (DEMENT* OR ALZHEIMER* OR "MAJOR NEUROCOGNITIVE DISORDER*") | 6,878 |
| 2 | (APP OR APPS OR "AUGMENTED REALIT*" OR "BLENDED TREATMENT" OR "BLENDED THERAPY" OR BLOG OR "CELL PHONE*" OR CELLPHONE* OR CHAT OR CHATS OR CHATBOT* OR COMPUTER* OR DIGITAL* OR "E COUNSELING" OR ECOUNSELING OR E-COUNSELING OR "E COUNSELLING" OR ECOUNSELLING OR E-COUNSELLING OR "E HEALTH" OR EHEALTH OR EHEALTH OR ELECTRONIC* OR EMAIL* OR E-MAIL* OR FACEBOOK OR ICT OR INTERNET OR MESSENGER OR MHEALTH OR M-HEALTH OR "MOBILE DEVICE" OR "MOBILE HEALTH" OR "MOBILE  PHONE" OR "MOBILE-HEALTH" OR ONLINE* OR PHONE OR REMOTE OR SKYPE OR SMARTPHONE* OR SMS OR "SOCIAL MEDIA" OR TABLET OR TABLETS OR TECHNOLOG* OR "TELE HOME CARE" OR "TELE MEDICINE" OR TELECARE OR TELE-CARE OR TELECARING OR TELE-CARING OR TELECOMMUNICATION OR TELECONFERENCE* OR TELE-CONFERENCE* OR TELECONSULTATION* OR TELE-CONSULTATION* OR TELEHEALTH OR TELE-HEALTH OR TELEMEDICINE OR TELENURSE OR TELENURSING OR TELEPHONE OR TELEPHONES OR TELEPRESENCE OR TELEPRESENT OR TELEREHABILITATION OR TELE-REHABILITATION OR TELETHERAP* OR TELE-THERAP* OR TELETREATMENT OR TELE-TREATMENT OR VIDEOCONFERENCE OR VIDEOCONFERENCES OR VIRTUAL OR "WEB BASED" OR WEBBASED OR WEB-BASED OR "WORLD WIDE WEB") | 384,401 |
| 3 | (ADVICE* OR ADVISE* OR ADVISING OR COACH* OR CONSULT* OR COUNSELLING OR COUNSELING OR INSTRUCT* OR GUIDANCE* OR GUIDE* OR MENTORING OR "PASTORAL CARE" OR "SOCIAL SUPPORT*" OR SUPERVIS* OR "SUPPORT SERVICE*" OR "TALKING THERAP*") | 63,379 |
| 4 | 1 AND 2 AND 3 | 1,148 |
| 5 | (SURGICAL OR SURGERY OR GENETI* OR PROTEOMIC* OR GENOMIC* OR PROTEIN* OR NEUROIMAG*) | 106,219 |
| 6 | 4 NOT 5 | 967 |

| **Web of Science Core Collection**  Date: December 08, 2023  Filter: 2021-2023 | | |
| --- | --- | --- |
| **#** | **Entry** | **Hits** |
| 1 | TS=(DEMENT* OR ALZHEIMER* OR "MAJOR NEUROCOGNITIVE DISORDER*") | 74,572 |
| 2 | TS=(APP OR APPS OR "AUGMENTED REALIT*" OR "BLENDED TREATMENT" OR "BLENDED THERAPY" OR BLOG OR "CELL PHONE*" OR CELLPHONE* OR CHAT OR CHATS OR CHATBOT* OR COMPUTER* OR DIGITAL* OR "E COUNSELING" OR ecounseling OR E-COUNSELING OR "E COUNSELLING" OR ecounseling OR E-COUNSELLING OR "E HEALTH" OR EHEALTH OR E-HEALTH OR ELECTRONIC* OR EMAIL* OR E-MAIL* OR FACEBOOK OR ICT OR INTERNET OR MESSENGER OR MHEALTH OR MHEALTH OR "MOBILE DEVICE" OR "MOBILE HEALTH" OR "MOBILE PHONE" OR "MOBILE-HEALTH" OR ONLINE* OR PHONE OR REMOTE OR SKYPE OR SMARTPHONE* OR SMS OR "SOCIAL MEDIA" OR TABLET OR TABLETS OR TECHNOLOG* OR "TELE HOME CARE" OR "TELE MEDICINE" OR TELECARE OR TELE-CARE OR telecarding OR TELE-CARING OR TELECOMMUNICATION OR TELECONFERENCE* OR TELE-CONFERENCE* OR TELECONSULTATION* OR TELE-CONSULTATION* OR TELEHEALTH OR TELE-HEALTH OR TELEMEDICINE OR telenurses OR TELENURSING OR TELEPHONE OR TELEPHONES OR TELEPRESENCE OR telepresence OR TELEREHABILITATION OR TELE-REHABILITATION OR TELETHERAP* OR TELE-THERAP* OR teletreatments OR teletreatments OR VIDEOCONFERENCE OR VIDEOCONFERENCES OR VIRTUAL OR "WEB BASED" OR WEBBASED OR WEB-BASED OR "WORLD WIDE WEB") | 1,234,647 |
| 3 | TS=(ADVICE* OR ADVISE* OR ADVISING OR COACH* OR CONSULT* OR COUNSELLING OR COUNSELING OR INSTRUCT* OR GUIDANCE* OR GUIDE* OR MENTORING OR "PASTORAL CARE" OR "SOCIAL SUPPORT*" OR SUPERVIS* OR "SUPPORT SERVICE*" OR "TALKING THERAP*") | 585,192 |
| 4 | 1 AND 2 AND 3 | 1.393 |
| 5 | TS=(SURGICAL OR SURGERY OR GENETI* OR PROTEOMIC* OR GENOMIC* OR PROTEIN* OR NEUROIMAG*) | 1,254,190 |
| 6 | 4 NOT 5 | 1,161 |

| **PsycINFO via Ovid**  Date: December 08, 2023  Filter: 2021 -Current | | |
| --- | --- | --- |
| **#** | **Entry** | **Hits** |
| 1 | (DEMENT* or ALZHEIMER* or "MAJOR NEUROCOGNITIVE DISORDER*").ti,ab,mp. or exp DEMENTIA/ or exp ALZHEIMER DISEASE/ | 16080 |
| 2 | (APP OR APPS OR "AUGMENTED REALIT*" OR "BLENDED TREATMENT" OR "BLENDED THERAPY" OR BLOG OR "CELL PHONE*" OR CELLPHONE* OR CHAT OR CHATS OR CHATBOT* OR COMPUTER* OR DIGITAL* OR "E COUNSELING" OR ECOUNSELING OR E-COUNSELING OR "E COUNSELLING" OR ECOUNSELLING OR E-COUNSELLING OR "E HEALTH" OR EHEALTH OR E-HEALTH OR ELECTRONIC* OR EMAIL* OR E-MAIL* OR FACEBOOK OR ICT OR INTERNET OR MESSENGER OR MHEALTH OR MHEALTH OR "MOBILE DEVICE" OR "MOBILE HEALTH" OR "MOBILE PHONE" OR "MOBILE-HEALTH" OR ONLINE* OR PHONE OR REMOTE OR SKYPE OR SMARTPHONE* OR SMS OR "SOCIAL MEDIA" OR TABLET OR TABLETS OR TECHNOLOG* OR "TELE HOME CARE" OR "TELE MEDICINE" OR TELECARE OR TELE-CARE OR TELECARING OR TELE-CARING OR TELECOMMUNICATION OR TELECONFERENCE* OR TELE-CONFERENCE* OR TELECONSULTATION* OR TELE- CONSULTATION* OR TELEHEALTH OR TELE-HEALTH OR TELEMEDICINE OR TELENURSE OR TELENURSING OR TELEPHONE OR TELEPHONES OR TELEPRESENCE OR TELEPRESENT OR TELEREHABILITATION OR TELE-REHABILITATION OR TELETHERAP* OR TELE-THERAP* OR TELETREATMENT OR TELETREATMENT OR VIDEOCONFERENCE OR VIDEOCONFERENCES OR VIRTUAL OR "WEB BASED" ORWEBBASED OR WEB-BASED OR "WORLD WIDE WEB").TI,AB,MP. OR EXP TELEMEDICINE/ OR EXP TECHNOLOGY/ OR EXP VIRTUAL REALITY/ OR EXP TELECONFERENCING/ OR EXP ONLINE THERAPY/OR EXP TELEREHABILITATION/ OR EXP SOCIAL MEDIA/ OR EXP MOBILE DEVICES/ | 110,584 |
| 3 | (ADVICE* or ADVISE* or ADVISING or COACH* or CONSULT* or COUNSELLING or COUNSELING or INSTRUCT* or GUIDANCE* or GUIDE* or MENTORING or "PASTORAL CARE" or "SOCIAL SUPPORT*" or SUPERVIS* or "SUPPORT SERVICE*" or "TALKING THERAP*").ti,ab,mp. or exp COUNSELING/ or exp SOCIAL SUPPORT/ | 92,175 |
| 4 | 1 AND 2 AND 3 | 428 |
| 5 | (SURGICAL or SURGERY or GENETI* or PROTEOMIC* or GENOMIC* or PROTEIN* or NEUROIMAG*).mp. [mp=title, abstract, heading word, table of contents, key concepts, original title, tests & measures, mesh word] | 338,323 |
| 6 | 4 NOT 5 | 388 |

## **Free web search Google**

| **Search engine: Google** | | | |
| --- | --- | --- | --- |
| **Date: 18.04.2024** | **Language: any** | **Search filters: no** | **Number of references screened: first 40** |
| **Search terms** | | **Eligible references** | |
| counselling dementia technology | | - | |
| counseling dementia technology | | - | |
| counselling dementia ict | | - | |
| counseling dementia ict | | - | |
| e counselling dementia | | - | |
| e counseling dementia | | - | |
| Alzheimer’s counselling technology | | - | |
| Alzheimer’s counseling technology | | - | |
| dementia counselling web-based | | - | |
| dementia counseling web-based | | - | |
| support intervention dementia technology | | - | |
| support intervention dementia web-based | | - | |
| telecounselling dementia | | - | |
| telecounseling dementia | | - | |
| coaching dementia technology | | - | |
| telephone-based counselling dementia | | - | |
| telephone-based counseling dementia | | - | |

**Free web search Google Scholar**

| **Search engine: Google Scholar** | | | |
| --- | --- | --- | --- |
| **Date: 17/18.04.2024** | **Language: any** | **Search filters: no** | **Number of references screened: first 20** |
| **Search terms** | | **Eligible references** | |
| counselling dementia technology | | - | |
| counseling dementia technology | | - | |
| counselling dementia ict | | - | |
| counseling dementia ict | | - | |
| e counselling dementia | | - | |
| e counselling dementia | | - | |
| Alzheimer’s counselling technology | | - | |
| Alzheimer’s counseling technology | | - | |
| dementia counselling web-based | | - | |
| dementia counseling web-based | | - | |
| support intervention dementia technology | | - | |
| support intervention dementia web-based | | - | |
| telecounselling dementia | | - | |
| telecounseling dementia | | - | |
| coaching dementia technology | | - | |
| telephone-based counselling dementia | | - | |
| telephone-based counseling dementia | | Thai, G. H., Rivette, S., Sharman, J., Epps, F., & Masoud, S. (2024). Dementia Caregiver Experiences: Insights From a Telephone-Based Support Program. Journal of Applied Gerontology, 0(0). https://doi.org/10.1177/07334648241234745 | |
