# Supplementary material for: Program Theory and Core Outcome Set Development for a Technology-Assisted Counseling Intervention in Dementia: Multimethods Study
Source: J Med Internet Res. 2026 Jan 20;28:e81669. doi: 10.2196/81669 (PMC12818504; doi:10.2196/81669)
Supplement: Multimedia Appendix 2 [file jmir-v28-e81669-s002.docx]

**Interview guide for semi-structured interviews - caregivers**

Can you please tell me about your living situation with regard to caring for your relative?

What experiences you had with counseling in relation to care?

What did or do you expect from counseling and persons delivering counseling?

What aspects have an impact on counseling?

What do you think should or can be achieved through counseling? How can this be achieved?

In your opinion, how can you measure whether counseling is helpful and has a supportive effect?

**Interview guide for semi-structured interviews - counselors**

Can you please describe your everyday work at your counseling centre?

What expectations do you have of your job?

What expectations do you have of persons seeking counseling?

What aspects have an impact on counseling?

What do you think should or can be achieved through counseling? How can this be achieved?

In your opinion, how can you measure whether counseling is helpful and has a supportive effect?
