# Supplementary material for: Program Theory and Core Outcome Set Development for a Technology-Assisted Counseling Intervention in Dementia: Multimethods Study
Source: J Med Internet Res. 2026 Jan 20;28:e81669. doi: 10.2196/81669 (PMC12818504; doi:10.2196/81669)
Supplement: Multimedia Appendix 3 [file jmir-v28-e81669-s003.docx]

## Table S1. Description of studies reporting on included intervention programmes.^a^

| **Intervention^b^** | **Reports included^c^**  **Country** | **Study design** | **Aim(s) of report** | **Number of participants / contacts** | **Outcomes caregiver**  **(assessment instrument)** | **Outcomes person with dementia**  **(assessment instrument)** |
| --- | --- | --- | --- | --- | --- | --- |
| Admiral Nurse Helpline | **Wilkinson 2016** [70]  UK | Quantitative descriptive and qualitative study | Analysis and evaluation of the intervention | n=305 contacts | N/A | N/A |
|  | Brown 2020a [93]  UK | N/A (practical report) | Exploring the use of telephone helplines during SARS-CoV-2 pandemic; Reflection on the experience of a dementia specialist helpline nurse | N/A |  |  |
|  | **Brown 2020b** [108]  UK | Case study | Presentation and reflection of a call to the Admiral Nurse Dementia Helpline | n=1 participant |  |  |
|  | **Drayton 2020** [94]  UK | Case study | Discussion of the role of managing complexity on a dementia telephone helpline | n=1 participant |  |  |
| ADS Helpline | Gilliard 1998 [71]  UK | Quantitative descriptive (letter to editor) | Brief description of project results | n=60 contacts | N/A | N/A |
| Alzheimer Helpline | Jansen 2007 [72]  Germany | Quantitative descriptive | Evaluation of helpline service (e.g., caller demographics, situation of persons with dementia, ways of access, topics discussed, satisfaction with service) | n=4,837 contacts | N/A | N/A |
|  | Pendergrass 2019 [73]  Germany | Quantitative descriptive | Evaluation of helpline service (e.g., caller demographics, topics discussed, ways of access) | n=3,744 participants |  |  |
|  | Kurz 2024 [74]  Germany | Quantitative descriptive | Evaluation of the extent to which the helpline service meets the quality criteria of telephone counselling and the expectations and needs of those seeking advice | n=201 participants | N/A | N/A |
| CANDID | Harvey 1998 [75]  UK | Quantitative descriptive | Audit and evaluation of the introduction of a novel support service | n=1,121 contacts | N/A | N/A |
| Care Consultation / Care Consultation Plus | Hodgson 2021 [41]  USA | RCT | Evaluation of the effectiveness of Helpline ‘Care Consultation’ and ‘Care Consultation Plus’ conditions | n=445 participants | Global Health (PROMIS Global Health: Global Physical Health (GPH) and Global Mental Health GMH))  Self-efficacy  (PROMIS Self-Efficacy for Emotions (derived from the Self Efficacy for Managing Chronic Disease (SEMCD) questionnaire)) | N/A |
| Coyne^b^ comparator / experimental | Coyne 1995 [42]  USA | RCT | Evaluation of the effectiveness of standard and extended helpline services | n=62 participants | Depression  (Zung Self-Rating Depression Scale)  Burden (Zarit Burden Interview ZBI)  Resource use  (Number of community services used) | Resource use (Number of community services used) |
| D-PACT | Wheat 2023 [83]  UK | Qualitative study | Examining multiple experiences and perceptions of remotely delivered care from persons with dementia, caregivers, practitioners, researchers | n=22 participants  (n=8 person with dementia / caregiver dyad; n=7 caregiver; n=2 persons with dementia; n=5 practitioners)  + data from n=11 non-participant observations | N/A | N/A |
| FITT-C | National Institutes of Health 2008 [44]  USA | N/A (registry entry) | N/A | N/A | Depression (Center for Epidemiology Studies Depression Scale CES-D)  Burden (Zarit Burden Interview ZBI)  Reaction to care-receiver behaviour (Revised Memory and Behavior Problem Checklist RMBPC)  Family functioning (Family Assessment Device FAD)  Self-efficacy (Self-Efficacy Questionnaire SEQ)  Positive aspects of caregiving (Positive Aspects of Caregiving scale PAC)  Quality of life (Euro Quality of Life Visual Analog Scale EQ-5D VAS)  Resource use  (Number of community and healthcare services used) | Resource use (Number of community and healthcare services used) |
|  | Tremont 2011 [45]  USA | RCT (conference abstract) | Exploration whether caregiver characteristics were related to credibility and expectancy; Examination whether these variables were related to caregivers’ early response to two interventions | n=133 participants |  |  |
|  | Tremont 2013a [46]  USA | RCT (conference abstract) | Examination of the efficacy of FITT-C for reducing distress | n=237 participants |  |  |
|  | **Tremont 2013b** [47]  USA | RCT (study protocol and baseline data) | Description of study design, methodology, and baseline data | n=250 participants |  |  |
|  | Tremont 2014 [48]  USA | RCT (conference abstract) | Examination of the effects of FITT-C on community support and healthcare use | n=250 participants |  |  |
|  | **Tremont 2015** [49]  USA | RCT | Examination of the effects of a telephone-based intervention on caregiver well-being | n=250 participants |  |  |
|  | **Tremont 2017** [50]  USA | RCT | Examination of the effects of a telephone-delivered intervention on community support and healthcare use | n=250 participants |  |  |
| FITT-D | Tremont 2008 [43]  USA | RCT | Examination of the preliminary effectiveness of FITT-D for reducing caregiver burden, depression, and reactions to memory and behaviour problems | n=60 participants | Depression  (Geriatric Depression Scale GDS)  Burden  (Zarit Burden Interview ZBI)  Reaction to care-receiver behaviour  (Revised Memory and Behavior Problem Checklist RMBPC)  Knowledge  (Alzheimer’s Disease Knowledge Test)  General health  (SF 36 General Health)  Self-efficacy  (Self Efficacy Scale SEQ)  Family functioning  (Family Assessment Device FAD)  Perceived social support  (Multidimensional Scale of Perceived Social Support MSPSS) | N/A |
| Helpline Alz Ass East Massa | Silverstein 1993 [76]  USA | Quantitative descriptive | Evaluation of helpline services (e.g., caller demographics, demographics of persons with dementia, ways to access, reasons for calling, information provided, satisfaction with service) | n=100 participants | N/A | N/A |
| Natale^b^ | Natale 2012 [66]  Italy | Non-randomized trial (letter to editor) | Evaluation of the impact on disease-management effectiveness | n=52 participants | Caregiver distress  (Caregiver Burden Inventory CBI) | N/A |
| NCSP | Rozani 2022 [77]  Israel | Quantitative descriptive (poster abstract) | Evaluation of callers’ characteristics and behaviours over time; Experience with provided integrated care, Satisfaction with service | n=11,820 calls  + n=376 home visits n=411 virtual visits | N/A | N/A |
| NVAMP | Chang 2004 [84]  USA | Qualitative study | Description of caregivers’ perceived reasons for helpfulness of telephone calls | n=83 participants | N/A | N/A |
| ODCC | Nakano 2018 [78]  Japan | Quantitative descriptive | Evaluation of a telephone support system (e.g., callers demographics, demographics of persons with dementia, topics discussed, advice provided) | n=1,485 contacts | N/A | N/A |
| Salfi^b^ nonanonym / anonym | **Salfi 2004** [85]  Canada | Qualitative study (dissertation) | Exploring the intervention of telephone support | n=2 agencies providing telephone support  (n=8 callers and n = 4 provider participated) | N/A | N/A |
|  | **Salfi 2005** [86]  Canada | Qualitative study |  |  |  |  |
|  | Spilsbury 2006 [87]  Canada | Comment on Salfi 2005 | Reflection and discussion of study methodology and results |  |  |  |
| Admiral Nurse practice development project | Madden 2022 [88]  UK | Qualitative study (participatory action research) | Evaluation of an practice development project by reflecting on challenges and benefits of video consultations for caregivers | n=12 contacts | N/A | N/A |
| ALZ i-Connect | Tousi 2017 [79]  USA | Quantitative descriptive | Evaluation of a novel implemented audiovisual care consultation | n=11 participants | N/A | N/A |
| TeleFAMILIES | Rice 2022 [69]  USA | Quantitative pre- and post-measures | Detecting statistically significant changes in caregiver burden, depressive symptoms, caregivers’ ability to cope and manage changed behaviour of persons with dementia; Comparing effectiveness of telehealth and in-person administration of the intervention | n=216 participants (n = 59 telehalth,  n = 157 in-person) | Burden  (Zarit Burden Interview ZBI)  Depression  (Center for Epidemiologic Studies Depression Scale-Revised CESD-R)  Reaction to care-receiver behaviour  (Revised Memory and Behavior Problems Checklist RMBPC) | N/A |
| Sabat^b^ | Sabat 2011 [95]  USA | Case study | Examination of the effect of email education, counselling, and psychosocial support on the caregiver’s evolving subjective experience | n=1 participant (1,276 contacts) | N/A | N/A |
| CuidaTEXT | National Institutes of Health 2020 [104]  USA | N/A (registry entry) | N/A | N/A | Behavioural symptom severity  (Neuropsychiatric Inventory-Questionnaire NPI-Q Distress)  Caregiver strain  (Modified Caregiver Strain Index CSI)  Burden  (Zarit Burden Interview ZBI-6)  Caregiving competence (Preparedness for Caregiving Scale PCS)  Positive aspects of caregiving (Positive aspects of caregiving scale PAC)  Unmet needs  (Measure of Unmet Needs UN)  Dementia knowledge (Epidemiology/Etiology Disease Scale EEDS)  Social support  (Interpersonal Support Evaluation List ISEL-12)  Coping  (Coping Orientation to Problems Experienced Inventory COPE-28)  Depression  (Center for Epidemiologic Studies Depression Scale CES-D-10)  Affect  (Scale of Positive and Negative Experience SPANE)  Self-perceived health  (one-item question) | Instrumental activities of daily living  (Functional Activities Questionnaire FAQ)  Behavioural symptom severity  (Neuropsychiatric Inventory-Questionnaire NPI-Q Severity) |
|  | Puchalt 2020 [105]  USA | N/A (project presentation, conference abstract) | Description of the CuidaTXT Project | N/A |  |  |
|  | Perales-Puchalt 2022 [98]  USA | Mixed methods study | Describing the development of a tailored SMS text messaging intervention to support Latinx family caregivers of persons with dementia | n=6 caregivers and n=16 health professionals as study advisory board members;  n=5 participants in usability testing |  |  |
|  | **Perales-Puchalt 2024** [106]  USA | Pilot and feasibility study (pre-post-intervention trial) | Testing feasibility, acceptability, and preliminary efficacy of a bidirectional text message intervention to support Latino dementia family caregivers | n=24 participants |  |  |
| STAV | Dorell 2022 [89]  Sweden | Qualitative study | Description of the experiences of family caregivers to persons with dementia when receiving professional support via an interactive mobile app | n=12 participants | N/A | N/A |
| ICSS | Chui 2005 [90]  Canada | Qualitative study (conference abstract) | Reporting on the developmental phase of a new internet-based support services | n=3 participants | Burden  (Burden Scale for Family Caregivers BSFC) | N/A |
|  | **Chui 2008** [99]  Canada | Mixed methods study (dissertation) | Description and explanation (non-)usage behaviour of e-health services among caregivers | n=46 participants |  |  |
|  | **Chui 2009** [100]  Canada | Mixed methods study | Assessment of the usability of a new Internet-based Caregiver Support Service  Evaluation of the effects on health outcomes of caregivers | n=28 participants |  |  |
|  | Chui 2010a [101]  Canada | Mixed methods study | Explore the usage behaviour of users of e-health services  Exploration of factors (predictors) associated with uptake and use of an internet-mediated intervention for caregivers | n=46 participants |  |  |
|  | Chui 2010b [91]  Canada | Qualitative study | Analysis of the content and process of the new intervention  Explore how the client-centred concepts can be applied to Internet-mediated intervention | n=28 participants |  |  |
|  | Chui 2011 [92]  Canada | Qualitative study | Improvement of understanding of family caregivers’ use of Web-based intervention support | n=14 participants |  |  |
| InformCare | **Barbabella 2016** [102]  Italy, Sweden, Germany | Mixed methods study | Examination of the impact of the Web-based psychosocial intervention on caregivers | n=123 participants | Psychological well-being  (World Health Organization Well-being Index WHO-5)  Self-perception of negative and positive aspects of caregiving  (Carers of Older People in Europe COPE Index)  Social support  (Multidimensional Scale of Perceived Social Support MSPSS) | N/A |
|  | Lamura 2017 [107]  Italy, Sweden, Germany | N/A (project report) | Description of the platform content, the piloting and implementation process, critical reflections and sustainability | N/A |  |  |
|  | **Barbabella 2018** [103]  Italy, Sweden, Germany | Mixed methods study | Report on the usage and usability evaluation of a multicomponent Web-based programme for psychosocial support for family caregivers | n=123 participants |  |  |
| Link2Care | Kelly 2003 [80]  USA | Quantitative descriptive | Report on users’ demographics and satisfaction | N/A | N/A | N/A |
| Online Coaching | Rentz 2010 [81]  USA | Quantitative descriptive | Report on utilisation rates and users’ satisfaction | n=121 participants | N/A | N/A |
| De Cola^b^ | De Cola 2016 [82]  Italy | Quantitative descriptive | Evaluation of usability and effects of the telehealth system on caregivers and persons with dementia | n=38 participants | Burden  (Caregiver Burden Inventory CBI) | Global cognitive state  (Mini-Mental State Examination MMSE)  Functional dependency (Activities of Daily Living ADL and Instrumental Activities of Daily Living Scale IADL)  Depression  (Geriatric Depression Scale GDS)  Cognitive impairment  (Bedford Alzheimer Nursing Severity Scale BANSS)  Psychiatric symptoms  (Brief Psychiatric Rating Scale BPRS) |
| Laver^b^ | Australian New Zealand Clinical Trials Registry 2017 [51]  Australia | N/A (registry entry) | N/A | N/A | Mastery  (Caregiving Mastery Index CMI)  Perceived change  (Perceived Change Scale PCS)  Upset  (Caregiver Behavioral Occurrence and Upset Scale (modeled on the Agitated Behavior in Dementia Scale)) | Functionality  (Caregiver Assessment of Function and Upset CAFU)  Number of behaviours such as verbal aggression, refusing care, restlessness, anxiety, waking overnight, and repetitive questioning  (Caregiver Behavioral Occurrence and Upset Scale (modeled on the Agitated Behavior in Dementia Scale)) |
|  | **Laver 2020** [52]  Australia | RCT | Determination whether delivery of a dyadic intervention using telehealth was noninferior to delivery of the same programme using home visits | n=63 dyads |  |  |
|  | Laver 2021 [53]  Australia | N/A (abstract podium presentation) | Share experiences and tips on telehealth delivery for people with dementia and their caregivers | N/A |  |  |
| RCTM | National Institutes of Health 2016 [54]  USA | N/A (registry entry) | N/A | N/A | Subjective stress  (seven-item measure of  care-related strain, one-item measure assessing the caregiver’sdifficulty with the relative’s mental or emotional state, adapted Zarit Burden Interview ZBI)  Depressive symptoms  (Center for Epidemiological Studies-Depression Scale CES-D, Mood Assessment Scale MAD)  Secondary role strains  (two single-item ratings of the caregiver’s and the relative’s adjustment to RLTC placement)  Residential care stress  (six-item measure for perceptions of staff communication with family, five-item measure for staff support for family, 10-item measure assessing five positive and five negative types of caregiver interactions with their relative, staff, other family, Item caregivers’ upset to see their relative in a residential care setting, 28-item Family Involvement Interview)  Sense of capability  (Short Sense of Competence  Questionnaire SSCQ)  Caregiver SEQ  (Self-Efficacy Questionnaire SEQ) | N/A |
|  | Gaugler 2020 [55]  USA | N/A (study protocol) | N/A | N/A |  |  |
|  | **Statz 2022** [56]  USA | Mixed methods study | Identification of areas of guilt experienced by caregivers of persons with dementia after long-term care transition; Exploration of caregivers’ characteristics predicting heightened feelings of guilt; Identification of how a counselling intervention can provide practical support to help alleviate caregivers’ guilt following transition | n=83 participants |  |  |
|  | Zmora 2021 [57]  USA | Mixed methods study | Improvement of understanding of the interpersonal and contextual factors that influence caregiver-staff relationships in residential long-term care facilities; Identification of targets for future interventions to improve relationships | n=85 participants |  |  |
|  | Zmora 2021 [58]  USA | N/A (corrigendum) | N/A | N/A |  |  |
|  | Albers 2023 [59]  USA | Qualitative study | Understanding how and why a multi-component intervention for caregivers of cognitively impaired relatives living in RLTC was perceived as beneficial, useful, and effective by participants | n= 30 |  |  |
|  | **Gaugler 2024** [60]  USA | RCT | Evaluating the efficacy of a psychoeducational and psychosocial support telehealth programme for family caregivers who have admitted a cognitively impaired relative to an RLTC setting | n=240 |  |  |
|  | Australian New Zealand Clinical Trials Registry 2021 [64] Australia | N/A (registry entry) | N/A | N/A | Perceived stress  (Perceived Stress Scale PSS)  Grief  (Caregiver Grief Scale CGS)  Depression  (Centre for Epidemiologic Studies Depression Scale CES-D)  Guilt  (Caregiver Guilt Questionnaire CGQ)  Anxiety  (Geriatric Anxiety Inventory GAI)  Support for caring  (Support for Caring subscale of the Adult Carer Quality of Life Questionnaire AC-QoL) | N/A |
|  | **Brooks 2024** [65]  Australia | Pilot and feasibility study (small-scale RCT + qualitative study) | Testing feasibility of participant recruitment and retention, feasibility of intervention delivery and data collection, acceptability of the intervention to carergivers, preliminary effects on caregiver perceived stress, anxiety, guilt, depression, grief, socio-emotional support | n=18 (RCT)  n=15 (qualitative study) |  |  |
| Twaddle^b^ | Twaddle 2021 [109]  USA (Pacific island of Guam) | N/A (practical report, podium presentation abstract) | Description of a telehealth outreach programme | N/A | N/A | N/A |
| Dementelcoach | van Mierlo 2011 [67]  The Netherlands | Non-randomized trial (conference abstract) | Evaluation of the impact of the intervention on family caregivers and trained telecoaches  Investigation of the effect of the intervention on the professional caregiver’s work satisfaction, work experience and self-esteem | N/A | Sense of capability  (Short Sense of Competence Scale SSCQ)  Mental health  (General Health Questionnaire GHQ-28)  Emotional impact of neuropsychiatric symptoms  (Neuropsychiatric Inventory NPI Burden subscale)  Quality of life and happiness  (two TOPICS-MDS items) | Neuropsychiatric symptoms  (Neuropsychiatric Inventory NPI Total symptoms and Symptoms severity) |
|  | **van Mierlo 2012** [68]  The Netherlands | Non-randomized trial | Evaluation of the effectiveness of telephone coaching on burden and mental health problems of family caregivers | n=54 participants |  |  |
|  | Netherlands Trial Register 2015 [61]  The Netherlands | N/A (registy entry) | N/A | N/A |  |  |
|  | Droes 2019a [62]  The Netherlands | RCT (conference abstract) | Evaluation of the feasibility, implementation and (cost-) effectiveness of the individualized Meeting Centers Support Program (iMCSP) | N/A |  |  |
|  | **Droes 2019b** [63]  The Netherlands | RCT | Exploration of the effectiveness of the individualized Meeting Centers Support Program (iMCSP) consisting of DemenTalent, Dementelcoach (telephone coaching), and STAR e-Learning for caregivers, compared to regular MCSP and No day care support. | N/A |  |  |
|  | **Van Rijn 2020** [96]  The Netherlands | Process evaluation | Identification of facilitating and impeding factors of linking Dementelcoach and STAR e-Learning to existing Meeting Centres for persons with dementia and their family caregivers | n=15 participants |  |  |
| Nomura^b^ | Nomura 2009 [97]  Japan | Process evaluation | Description of the implementation and process evaluation of the intervention | n=68 participants | N/A | N/A |

*Abbreviations*: ADS: Alzheimer's Disease Society; CANDID: Counselling and Diagnosis in Dementia; COACH: Comprehensive Older Adult and Caregiver Help; D-PACT: Dementia - PersonAlised Care Team; FITT-C: Family Intervention: Telephone Tracking – Caregiver; FITT-D: Family Intervention: Telephone Tracking – Dementia; Helpline Alz Ass East Massa: Helpline of the Alzheimer's Association of Eastern Massachusetts; ICSS: Internet-based Caregiver Support Service; N/A: not applicable/not available; NCSP: National Call Support Program for Alzheimer’s patients and their caregivers; NVAMP: Nurse Video With Assisted Modeling Program; ODCC: Okayama Dementia Call Center; RCT: Randomized controlled trial; RCTM: Residential Care Transition Module; RLTC: residential long-term care; STAV: Support for family caregivers (“STöd för AnhörigVårdare”); TeleFAMILIES: telehealth-administered Families Access to Memory Impairment and Loss Information, Engagement, and Supports; TOPICS-MDS: The Older Persons and Informal Caregivers Survey Minimum DataSet

*Notes*: ^a^ Fields marked in gray have been published previously [8].

^b^ When no name is reported, the name of the first author was assigned to the intervention.

^c^ If several publications on an intervention have been identified through the systematic literature search, main publications are marked in bold type, other
reports are considered as related publications.

## Table S2. Description of included intervention programmes.^a^

## By applying items of the Template for Intervention Description and Replication checklist (TIDieR) [30] and the revised Criteria for Reporting the Development and Evaluation of Complex Interventions in healthcare guideline (CReDECI 2) [31]

| **Intervention**  **(References)** | **Type of intervention (technology used)** | **Objective(s)** | **Theoretical underpinning** | **Components**  **(rationale, interaction)**  CReDECI 2 items 2, 3 | | **Provider**  (expertise, background,  specific training) | **Consumer** | **Duration/**  **Frequency/**  **Period** | **Implementation strategy (ImS)**  **Modifications of interventions (M)**  **Implementation barriers (B)**  **Implementation facilitators (F)**  **External conditions (ExtCon)** |
| --- | --- | --- | --- | --- | --- | --- | --- | --- | --- |
|  |  |  |  | **Procedures** | **Material** |  |  |  |  |
| TIDieR item 1 | TIDieR item 6 | TIDieR item 2 | CReDECI 2 item 1 | TIDieR item 4 | TIDieR item 3 | TIDieR item 5 |  | TIDieR item 8 | CReDECI 2 items 7, 11, 12  TIDieR item 10 |
| Admiral Nurse Helpline  [70,93,94,108] | Counselling via telephone (follow-up information by email or by post) | Providing specialist support as informants, educators and role models;  Helping families to cope with dementia | Core competencies required in telephone and e-counselling, which underpin the helpline approach (identified by the British Association for Counselling and and Psychotherapy) | Biopsychosocial assessment (Admiral Nurse assessment framework), checking out, reframing, and recapping on the conversation; Establishing rapport, emphasising, normalising, guided discovery, Socratic questioning; Giving advice; Balancing the needs of the person with dementia and the caller; Providing emotional support: understanding and empathy; Case management and coordination | Script proposal or suggested script (an example of what caregiver might say in a given situation) | Trained nurses with expertise in dementia care | Family and professional caregivers;  Persons with dementia | Calls often > 30 min  N/A  N/A | **ImS**: N/A  **M**: N/A  **B**: N/A  **F**: N/A  **ExtCond**: Helpline is part of Dementia UK |
| ADS  [71] | Counselling via telephone | Providing support, advice, and information | N/A | N/A | N/A | N/A | Family caregivers; Persons with dementia | x̅ = 6.3 min  N/A  N/A | **ImS**: N/A  **M**: Adapting helpline services to calls from persons with dementia  **B**: Uncertainty about counsellors’ role  **F**: Specific training and ongoing support of counsellors  **ExtCond**: Established helpline of the Alzheimer's Disease Society adapting to changing callers |
| Alzheimer Helpline  [72-74] | Counselling via telephone and email | Counselling, providing information, referral | N/A | N/A | Databases on dementia knowledge (literature) and local healthcare services for referral; Booklets | Professionals with qualifications in social work, social science, psychology, nursing, mental health, some also have experiences as family caregivers | Family and professional caregivers; Persons with dementia | x̅ = 11 min [6]  x̅ = 15 min [7]  x̅ = 29 min [8]  N/A  N/A | **ImS**: N/A  **M**: N/A  **B**: N/A  **F**: Regular team meetings, continuous supervision, external training; Easily accessible and free opportunity for getting support  **ExtCond**: Established helpline of the German Alzheimer Association |
| CANDID  [75] | Counselling via telephone (alternative: also via email, in person or by post) | Providing a point of contact and information for persons with young onset dementia and caregivers;  Providing clinical management advice and guidance to GPs | N/A | Collection of general information on callers for the CANDID database record; General information; Clinical advice (diagnosis, treatment, genetics) and social care advice | Activity reports, summaries of problems and advice; Letters sent to callers and/or General Practitioners | Trained nurses/counsellors (reviewed by a consultant neurologist and a psychiatrist) | Persons with dementia; Family and professional caregivers | N/A  N/A  N/A | **ImS**: N/A  **M**: N/A  **B**: N/A  **F**: Utilisation of a undemanding technology  **ExtCond**: Intervention is located at a specialist clinic |
| Care Consultation  [41] | Counselling via telephone | Providing emotional support, information and referrals to additional resources in the local community | N/A | Assessment of the caller’s situation; Provision of in-depth education about the disease and problem solving; Development of an action plan | Documentation | Care consultant with master’s degree in social work, counselling or related field | Family caregivers | N/A  N/A  N/A | **ImS**: N/A  **M**: N/A  **B**: N/A  **F**: Specific training of counsellors  **ExtCond**: Alzheimer’s Association National Helpline |
| Care Consultation Plus  [41] | Counselling via telephone | Providing emotional support, information and referrals to additional resources in the local community | N/A | Assessment of the caller’s situation; Provision of in-depth education about the disease and problem solving; Development of an action plan; Follow up to reinforce specific action steps and identify barriers to following through on the action steps | Documentation; Notes | Care consultant with master’s degree in social work, counselling or related field | Family caregivers | N/A  Initial + follow up call  1 month^d^ | **ImS**: N/A  **M**: N/A  **B**: N/A  **F**: Specific training of counsellors  **ExtCond**: Extended services of the Alzheimer’s Association National Helpline |
| Coyne^b^ comparator  [42] | Counselling via telephone | Providing information, referral, education, and counselling | N/A | Caller was encouraged to talk about any dementia-related issues he or she was concerned about; Questions were answered and requests for advice responded to; Referrals to community- based services | N/A | Staff with extensive experience working with persons with dementia and family caregivers | Family caregivers | N/A  Once  N/A | **ImS**: N/A  **M**: N/A  **B**: N/A  **F**: N/A  **ExtCond**: Established helpline (programme of the University of Medicine and Dentistry of New Jersey) |
| Coyne^b^ experimental  [42] | Counselling via telephone | Providing information, referral, education, and counselling | N/A | Caller was encouraged to talk about any dementia-related issues he or she was concerned about; Questions were answered and requests for advice responded to; Referrals to community- based services; Follow-up calls with further discussion of the caregiving issues and the offer of additional information, advice, and literature | Literature | Staff with extensive experience working with persons with dementia and family caregivers | Family caregivers | N/A  Helpline call + biweekly follow-up calls  8 weeks | **ImS**: N/A  **M**: N/A  **B**: N/A  **F**: N/A  **ExtCond**: Extended services of an established helpline (programme of the University of Medicine and Dentistry of New Jersey) |
| D-PACT  [83] | Counselling via telephone or videoconferencing | Coaching-based intervention aiming at understanding of ‘what matters’ and engaging persons with dementia and caregivers in care planning and shared decision making | Programme theory | Identification of ‘what matters’ and links between areas of concern, co-develop a ‘shared plan’ to address matters of importance | Visual (written or pictorial) tools depicting potential areas that persons may wish to focus on (‘What matters to you’ tool); Co-developed document detailing what actions had been agreed upon (’Plan of action’); Practitioner manual | Dementia support workers (DSWs) experience in working with persons with dementia and supervisors with extensive experience of providing dementia care and support, case management and multi-disciplinary team working, embedded in General  Practice (GP) surgeries | Persons with dementia; Family caregivers | N/A  N/A  N/A | **ImS**: N/A  **M**: Intended face-to-face delivery had to be adapted to remote delivery due to pandemic-related restrictions  **B**: Lack of environmental stimuli and information indicating increased needs; Difficulties in sharing materials and tools due to lack of software functionality; Limitations in the extent of shared understanding that can be achieved remotely; Consumer providing limited  verbal information about their situation; Consumers’ tendency to downplay or omit trouble talk  **F**: Creating prompt notes on possible topics of importance helped remote conversations continue to flow naturally; Interactional strategies to advance understanding; Options to meet separately and disclose privately via e-mail  **ExtCond**: N/A |
| FITT-C  [44-50] | Counselling via telephone | Providing information about dementia, recommendations for resources, and emotional support to reduce depression and burden in dementia caregivers; Enhancing coping through active problem solving; Facilitating positive changes within the family system | McMaster Model of Family Functioning, transition theory, and Lazarus and Folkman's Stress and Coping Models | Initial stage  Orientation and psychoeducation: introducing material and reviewing information about dementia, and psychological, emotional, psychosocial, medical effects of caregiving; Second stage  Telephone follow-up contacts: identifying new problems, positive and negative changes in caregivers or patients, reviewing psychoeducational information for a particular situation, assistance in problem solving;  Protocol:  1) Introduction – purpose of call;  2) Assessment of current status – positive and negative changes;  3) Assessment of key areas – changes in key areas of functioning;  4) Review of other issues that could be problematic;  5) Intervention – support and assistance to problem solving and utilisation of family resources;  6) Continuing education –opportunity for caregivers to ask questions | Therapist manual; Educational and resource material; Letter containing a brief biography and photograph of the assigned therapist; Letter summarising areas of progress and encouraging the caregiver to continue to develop and use adaptive coping strategies at conclusion of intervention | Trained master’s level therapists (mental health counsellors, social workers, nurses) | Family caregivers | Initial 60 min, follow-up 15–30 min  16 telephone contacts  6 months | **ImS**: N/A  **M**: N/A  **B**: N/A  **F**: Specific training and group supervision for counsellors; Simplicity and widespread availability of the telephone  **ExtCond**: N/A |
| FITT-D  [43] | Counselling via telephone | Providing emotional support, directing caregivers to appropriate resources, encouraging caregivers to attend to their own physical, emotional and social needs, and teaching caregivers strategies to cope with ongoing problems | Theoretical underpinnings are based on psychosocial transition, transactional stress and coping, and a systems view of family functioning (i.e., McMaster Model of Family Functioning) | Initial stage  Orientation and psychoeducation: introducing material and reviewing information about dementia, and psychological, emotional, psychosocial, medical effects of caregiving; Second stage  Telephone follow-up contacts: identifying new problems, positive and negative changes in caregivers or patients, reviewing psychoeducational information for a particular situation, assistance in problem solving | Treatment manual and interventions guide; Binder containing local resource information and educational material from the Alzheimer’s Association | Trained master’s level therapists | Family caregivers | Initial 60 min, follow-up 15–30 min  23 telephone contacts  12 months | **ImS**: N/A  **M**: N/A  **B**: N/A  **F**: Specific training and supervision for counsellors; Simplicity and widespread availability of the telephone  **ExtCond**: N/A |
| Helpline Alz Ass East Massa  [76] | Counselling via telephone | Providing access to comprehensive services; Providing support to caregivers | N/A | N/A | Information material | Psychologist, trained volunteers | Family and professional caregivers | x̅ = 14 min  N/A  N/A | **ImS**: N/A  **M**: N/A  **B**: N/A  **F**: N/A  **ExtCond**: Established Helpline of the Alzheimer's Association of Eastern Massachusetts |
| Natale^b^  [66] | Counselling via telephone | Improving disease management | N/A | Cousellors were available for questions, focusing on education‐behaviour problem‐solving strategies and practical advices | N/A | Neuropsychologist, trained geriatrician | Family caregivers | N/A  Monthly  6 months | **ImS**: N/A  **M**: N/A  **B**: N/A  **F**: N/A  **ExtCond**: Programme of a dementia clinic |
| NCSP  [77] | Counselling via telephone | Supporting caregivers of persons with dementia | N/A | Consul and practical tools addressing coping with daily medical/nursing-related tasks, managing behavioural and psychological symptoms of dementia, coping with their emotional burden, execution of rights | N/A | Multi-professional team of nurses and social workers | Family caregivers | N/A  N/A  N/A | **ImS**: N/A  **M**: N/A  **B**: N/A  **F**: National call support programme accessible 24/7  **ExtCond**: Three types of integrated care: single call support, call consultation combined with home visits, call consultation combined with a home visit and day care service |
| NVAMP  [84] | Counselling via telephone | Providing support for caregivers | N/A | 1) Initial 20-minute videotape providing information on promoting self-care abilities in dressing and eating for individuals with dementia;  2) Semi-structured telephone interviews identifying the concern or problem, sources of difficulty, possible sources of support, and alternatives for addressing problems; Nurses then conversed with caregivers about the various options and the pros and cons of the options available to them. Caregivers were assisted in reframing their concerns where possibilities were appropriate. Related behavioural disorders such as resistive and aggressive behaviour and incontinence, when brought up by caregivers, were discussed according to the problemsolving guidelines. | Videotape | Trained master’s level gerontological clinical nurse specialists | Family caregivers | x̅ = 18 min  Biweekly calls  12 weeks | **ImS**: N/A  **M**: N/A  **B**: N/A  **F**: Specific training for counsellors; Low-cost telephone support  **ExtCond**: N/A |
| ODCC  [78] | Counselling via telephone | Listening to the problems of clients and caregivers; Providing with useful information; Advising on necessary medical consultations | N/A | N/A | N/A | Experienced experts (family or public caregivers) | Persons with dementia; Family caregivers | x̅ = 20 min  N/A  N/A | **ImS**: N/A  **M**: N/A  **B**: N/A  **F**: N/A  **ExtCond**: Established helpline of the Okayama Dementia Call Center (ODCC) |
| Salfi^b^ anonym  [85-87] | Counselling via telephone | Providing confidential and caregiver-initiated telephone support | N/A | N/A | N/A | Health professionals with dementia training | Family caregivers | N/A  N/A  N/A | **ImS**: N/A  **M**: N/A  **B**: Loss of context associated with a telephone interaction; Not being able to follow up or call back  **F**: Convenience of telephone support  **ExtCond**: Established helpline of a branch of the Alzheimer Society |
| Admiral Nurse practice development project  [88] | Counselling via videoconferencing | Increasing accessibility and efficiency by remote delivery of psychosocial support for families affected by dementia | N/A | Initial assessment with offer to pursue contact via videoconferencing | Videoconferencing equipment | Trained nurses with expertise in dementia care | Family caregivers | N/A  N/A  N/A | **ImS**: N/A  **M**: Originally planned initial face-to-face assessment was carried out via videoconferencing due to pandemic-related restrictions  **B**: Suboptimal technology infrastructure, lack of internet access in remote areas and instable connectivity; Staff’s perceived lack of skills and confidence in setting up videoconferencing; Limited access to IT support due to pandemic-related restrictions; Concerns about confidentiality when working from home; Lack of prompts from the surroundings to keep the conversation going and encourage disclosures; Staff’s concerns about seing themselves on screen; Time constraints  **F**: Visual contact helped staff to focus more  **ExtCond**: Support by a practice development team, Embedded in the organisation’s strategy on learning and transformation; Overall impact of the pandemic |
| ALZ i-Connect  [79] | Counselling via videoconferencing | Providing the person with dementia and the family a welcoming and concerned person to introduce them to the services and supports available, connection of families; Giving information | N/A | Welcoming,  Introducing services of the Association; Coordinating a follow-up time to speak | Videoconferencing equipment | Helpline staff and care consultants with a bachelor’s or master’s degree in social work, counselling, or related degree | Persons with dementia and their families | N/A  N/A  N/A | **ImS**: N/A  **M**: N/A  **B**: Decreasing of nonclinical spaces; Technical challenges due to security issues (firewall)  **F**: “Face-to-face” conversation for first contact via videoconferencing software  **ExtCond**: Collaboration between staff of Memory Assessment Clinics and helpline staff of Alzheimer’s Association area chapter |
| TeleFAMILIES  [69] | Counselling via videoconferencing | Reducing caregiver burden by bolstering the support network, decreasing the emotional and practical demands and caregivers learning task-focused and non-avoidant coping skills | N/A | Semi-structured, person-centered assessment of caregivers’ physical, emotional, social needs, and current support network; Solution-focused counselling, targeting caregivers’ practical and emotional needs | N/A | N/A | Family caregiver | N/A  6 sessions (2 individual and 4 family counselling) + ad hoc counselling  6 months | **ImS**: N/A  **M**: N/A  **B**: N/A  **F**: n/A  **ExtCond**: Intervention bases on the New York University Caregiver Intervention (NYUCI) |
| Sabat^b^  [95] | Counselling via email | Providing education, counselling, and psychosocial support | Consumer’s experience is discussed in terms of Social Construction Theory and the idea of Locus of Control (LOC) | Information about aspects of care-receiver’s memory and selfhood that remained intact, how to interact with him to their mutual advantage, subjective experience of the care-receiver, and his reactions to, the losses he was experiencing, and how they affected his responses to the caregiver | N/A | Psychologist | Family caregiver | N/A  N/A  Approx. 3 years | **ImS**: N/A  **M**: N/A  **B**: N/A  **F**: N/A  **ExtCond**: N/A |
| CuidaTEXT [98, 104-106] | Counselling via SMS text messaging | Tailored SMS text messaging intervention to support Latinx family caregivers of individuals with dementia and address Latinx individuals’ disparities in access to caregiving support | Stress Process Framework;  Social Cognitive Theory | Live chat interaction with a coach for further help upon request | Reference booklet | Coach from the research team | Family caregivers | N/A  N/A  6 months | **ImS**: N/A  **M**: N/A  **B**: Mild technical issues  **F**: Text messaging is a well-suited modality to deliver caregiver support for Latinos; Interactions are enabled anytime thereby adressing Latino transportation barriers and incompatibilities of multiple care responsibilities; Available in English and Spanish using simple language thereby addressing language and literacy barriers; Deployed to caregivers’ private cellphones thereby addressing Latino caregivers’ shared family caregiving roles and dementia stigma  **ExtCond**: Counselling via SMS combined with daily automatic messages and keyword-driven text messages providing on-demand help |
| STAV  [89] | Counselling via an interactive mobile app combined with additional features | Supporting caregivers by encouraging dialogue with professionals, to take care of oneself, to get an overview of relevant information, to take notes in the diary to record challenging situations to discuss with professionals | N/A | N/A | N/A | Nurses | Family caregivers | N/A  N/A  8 weeks | **ImS**: N/A  **M**: N/A  **B**: Delayed or sporadic responses; Lack of continuity of contact nurses; Lack of information on chat partner; Lack of information on features; Technical difficulties  **F**: Possibility to have direct contact with a health professional at their own convenience and at times when needed  **ExtCond**: Counselling via chat combined with mindfulness exercises, weblinks to relevant sites, own contact list of services relevant to their caregiving tasks, personal diary |
| ICSS  [90-92,99-101] | Web-based psychosocial intervention: information, communication and counselling | Helping caregivers manage more effectively the burden of caring for a family member with dementia; Improvement the overall quality of life for the caregiver and the care recipient | Core concepts of client-centred practice;  Andersen’s Behavioral Model of Health Service Utilization (BMHSU), Venkatesh’s Unified Theory of Acceptance and Use of Technology (UTAUT), Eysenbach’s Law of Attrition (LOA), and Wilson’s and Chatman’s Models of Information Behaviour were used to explain ehealth usage behaviour | Personalised, asynchronous e-mail intervention for Chinese Canadian family caregivers in the language of choice via secure Web site; Therapists initiated contact, introduced themselves, asked each participant to share any concerns; Strategies such as empathic understanding, validation of emotions, and reactions to difficult caregiving situations, and informational and emotional support | Information website (caregiving information handbook with more than 400 pages); Enhanced functions (Family Forum, Short Stories, Telling More) | Experienced clinicians with a clinical background in occupational therapy and/or social work; staff were fluent in Chinese and could read and write Chinese | Family caregivers | N/A  N/A  6 months | **ImS**: N/A  **M**: Bi-weekly messages to increase awareness of the service; Functions and designs to enhance usability such as navigation aids  **B**: Problems in accessing accounts in an unfamiliar portal; Differences in the ability to express oneself in writing; Limited access to or lack of or insufficient hardware; Time constraints  **F**: Caregivers could sent an e-mail to an assigned therapist at a time that was convenient to them, using a language of their choice  **ExtCond**: Personalised e-mail support in combination with an information website containing over 400 pages of disease-specific, caregiving, and community resources information |
| InformCare  [102,103,107] | Web-based psychosocial intervention: information, communication and counselling | Offering information resources and interactive services to enable professional and peer support | N/A | Services delivered as individual and group online support provided in terms of information, advice, counselling, emotional and social support managed by a professional moderator via synchronous / asynchronous communication | Set of Web tools (social network, forum, private message feature, chat feature, videochat feature) to enable communication among caregivers and between caregivers and professionals; Guidelines for moderators; Guide for accessing and using the platform | Psychologist or social workers | Family caregivers | N/A  N/A  N/A | **ImS**: N/A  **M**: Adjustment of information resources and a revision of guidelines for implementing and moderating interactive services after piloting  **B**: Technical or usability issues as a reason for not having used the interactive services more; Lack of digital skills training for caregivers; Significant amount of ongoing support and investment demanded  **F**: Services provision customised to the sociocultural peculiarities and digital skills of national samples; Technical support guaranteed by the moderator  **ExtCond**: Web platform with interactive services areas and information resources (four main sections to improve knowledge and self-awareness concerning the caregiver’s role, coping strategies, support available) |
| Link2Care  [80] | Web-based psychosocial intervention: information, communication and counselling | Increase caregivers well-being and coping skills through convenient access to information, connection to other caregivers, and other services | N/A | N/A | N/A | Technical experts in caregiving, law, and health | Family caregivers | N/A  N/A  N/A | **ImS**: N/A  **M**: N/A  **B**: N/A  **F**: N/A  **ExtCond**: Communication via e-mail with a family consultant as part of a online programme with various features |
| Online Coaching Program  [81] | Web-based psychosocial intervention: information, communication and counselling | Providing individualized support and information to caregivers | N/A | Participants were assigned to a coach and communicated by e-mail, journal entries and occasional telephone conversations; Participants received a automated notification when the coach has sent a reply (mostly within 24 hours) | Information material | Social worker | Family caregivers | N/A  N/A  N/A | **ImS**: N/A  **M**: N/A  **B**: N/A  **F**: Combining the convenience and flexibility of internet support with the reliability and expertise of Alzheimer’s Association’s clinical staff; Complementing the more traditional helpline model while meeting the changing needs of the caregiver population  **ExtCond**: Programme of a chapter of the Alzheimer’s Association to be accessed via a secured personal member page including resource center with a library of hand-selected materials from the coach, journal section, health and wellbeing surveys, forums |
| De Cola^b^  [82] | Videoconference- or telephone-based counselling combined with tele-monitoring and/or psychoeducation | Providing a health care service for the elderly and support to their caregivers | N/A | N/A | Technological devices for videoconferencing and tele-monitoring | Neurologist, psychologist  (assistance by other health care professionals) | Persons with dementia; Family caregivers | x̅ = 30 min  Weekly  N/A | **ImS**: N/A  **M**: N/A  **B**: Care programme was provided by the local Government for only two years  **F**: Access was facilitated by a health care professional previously trained at the telemedicine centre  **ExtCond**: Part of a telehealth system with tele-monitoring of care-receivers’ vital parameters and caregiver support within a family-centred service provided by a local day centre |
| Laver^b^ comparator  [51-53] | Videoconference- or telephone-based counselling combined with tele-monitoring and/or psychoeducation | Improving problem-solving skills, education, and building skills; Addressing stress management; Working with the dyad to enhance activity engagement in the person with dementia | Adapted version of the COPE dyadic intervention as a theory-based intervention | Visits in-home and via videoconferencing software; In-home visits provided the opportunity for environmental assessment, rapport building and familiarity with videoconferencing programme; Assessment of the person with dementia, the caregiver and their environment followed by identification of key care challenges | Tablet with the videoconferencing software already installed if needed; Intervention manual; Treatment notes, time logs, records | Trained occupational therapists | Family caregivers; Persons with dementia | x̅ = 60 min  8 sessions (incl. 2 home visits)  16 weeks | **ImS**: N/A  **M**: N/A  **B**: N/A  **F**: Regular meetings with interventionists to discuss cases and treatment plans and monitor fidelity to the intervention; Access to an information technology telehealth specialist for troubleshooting  **ExtCond**: Face-to-face and telehealth delivery of the same dyadic intervention |
| RCTM  [54-60] | Videoconference- or telephone-based counselling combined with tele-monitoring and/or psychoeducation | Providing support and skills-building to help caregivers adapt to their care recipient’s transition to longterm care | Stress Process Model for Residential Care (SPM-RC) | Establish a therapeutic rapport with the caregiver and the family; Provide a safe environment to explore stressors; Examine family relational dynamics as they relate to the RLTC placement decision itself, as well as the roles different family members play in the life of the caregiver and relative in RLTC; Identify new modes of communication to facilitate more effective interactions with other family members and care staff; Identify effective ways to advocate for improved quality of care for and quality of life of their relatives in RLTC | Videoconferencing equipment; Treatment manual; Contact logs; Internet resources (links to websites, videos, articles); Printed resource materials; Training for interventionists | Designated trained transition counsellors (master’s level marriage and family therapist and interventionist with a PhD in clinical psychology with considerable counselling experience) | Family caregivers | x̅ = 81 min  6 sessions  4 months  + optional ad-hoc sessions over 12 months  + quarterly check-in calls | **ImS**: Treatment Delivery: Documentation of interventionist compliance to intended treatment and modifications; Treatment Receipt: Extent to which processes are implemented by participant and/or goals are met; Treatment Enactment: Extent to which knowledge and skills acquired during treatment are applied inreal world settings outside of treatment  **M**: N/A  **B**: N/A  **F**: N/A  **ExtCond**: N/A |
|  |  | Boosting the internal resources available to the carer (i.e., the mediating processes of cognitive appraisal, problem-focused coping, emotion-focused coping, support-seeking and acceptance) by providing emotional and practical support and with direction to other external resources as necessary | Expanded Stress Process Model of Family Caregiving in Institutional Settings |  |  |  |  |  |  |
| RCTM  [64,65] | Videoconference- or telephone-based counselling combined with tele-monitoring and/or psychoeducation | To ease the residential care transition process for families of persons with dementia and help family caregivers to better cope and adjust once their relative has been admitted into residential care | Expanded Stress Process Model of Family Caregiving in Institutional Settings | Processing the caregiver’s experience and helping to review and validate the decision to admit their relative into residential care; Assessment of individual needs and key issues; Problem-solving and coping strategies; Psychological and emotional validation and support; Direction to community support and resources | Standard PDF help sheet information (freely available from Dementia Australia); Intervention protocol | Trained Transition Counsellor (registered mental health counsellor with knowledge and experience of dementia and caregiving, and Australian residential aged care facilities, and with educational and experiential grounding in grief counselling and in stress management techniques) | Family caregivers | N/A  6 sessions  12 weeks  + Optional ad-hoc sessions | **ImS**: N/A  **M**: Cultural adaptation of the RCTM to an Australian context  **B**: Few technical issues  **F**: Delivery of the counselling sessions via videoconferencing was found to be acceptable, more convenient and comfortable than in-person sessions; Dose and spacing of sessions meet caregivers’ needs; Skills (empathy, non-judgmental) and dementia-specific knowledge of the Transition Counsellor  **ExtCond**: Time constraints of the pilot and feasibility study |
| Twaddle^b^  [109] | Videoconference- or telephone-based counselling combined with tele-monitoring and/or psychoeducation | Providing improved access to social support and specialised community  services for persons with dementia and their family caregivers | N/A | Telehealth family counselling employing narrative approaches that embrace the oral storytelling traditions of Pacific island cultures; 24/7 family caregiver support networks through mobile messaging apps to mitigate social isolation and ensure the availability of support in times of crisis | N/A | Dementia care specialists | Family caregivers; Persons with dementia | N/A  N/A  N/A | **ImS**: N/A  **M**: N/A  **B**: N/A  **F**: N/A  **ExtCond**: Counselling combined with access to virtual family caregiver support groups and virtual presentation and consultation sessions providing easy access to dementia care information and services |
| Dementel-coach  [61-63,67, 68,96] | Technology-based counselling as part of a comprehensive programme with non-technology-based components | Providing emotional, social, and practical support for caregivers; Increasing the support experience by caregivers and enhance their ability to cope with the consequences of dementia | Model of Determinants of Subjective Burden of Caregivers of Persons with Dementia based on the general stress-appraisal-coping theory of Lazarus and Folkman and the crisis model of Moos and Tsu | Caregivers were randomly assigned to a telephone coach | Training for telecoaches; Coach-to-coach sessions; Written practical guide | Trained professionals with experience in psychogeriatric care | Family caregivers | x̅ = 30-45 min  8-10 calls  20 weeks | **ImS**: Stepwise plan for implementation; Process evaluation based on the theoretical framework for tracing facilitators and barriers of adaptive implementation of care innovations  **M**: N/A  **B**: Lack of human and financial resources; Competing with other initiatives; Unexpected and impeding changes in organisation  **F**: Interest-holders found that the Dementelcoach concept was very clear to them; Support for implementation on organisational level; Organisation being located in an active region; Being part of a care and welfare network; Continuous promotion; Training on interventions to prepare for implementation; Commitment of management; Successful recruitment of participants; Collaboration with referrers; Added value for organisation  **B/F**: Laws and regulations; Policy of local government  **ExtCond**: Caregivers received telecoaching only or telecoaching in combination with day care; Telecoaching was further implemented in a comprehensive programme (individualized Meeting Centers Support Program consisting of DemenTalent (persons with dementia work as volunteers in a society based on their talents), Dementelcoach (telephone coaching), STAR e-Learning for caregivers, and day care) |
| Nomura^b^  [97] | Technology-based counselling as part of a comprehensive programme with non-technology-based components | Empowerment of persons with dementia to regain self-confidence and to improve relationships with families; Empowerment of caregivers to increase understanding of dementia, to educate on the appropriate use of social resources, to create a social network; Coaching of problem-focused coping; Improving or maintaining impaired cognitive function | Programme based on cognitive rehabilitation (concept originating from memory rehabilitation after brain injury) | Face-to-face and  phone counselling to coach problem-focused coping;  Strategies employed:  (1) commendation of the caregivers’ efforts, (2) identifying and focusing on one major problem, (3) explaining reasons behind the symptom or behaviour, (4) assuring caregivers’ feelings of upset and anger as natural reactions to the symptoms, (5) exploring specific strategies together | Log sheet to record content of counselling; Communication notebook | Trained social worker or public health nurses (certified care managers) | Family caregivers; Persons with dementia | N/A  monthly  N/A | **ImS**: N/A  **M**: N/A  **B**: Difficulty to recruit eligible persons and to explain the benefits of the research to family caregivers; Difficulties in dealing with persons with dementia with aphasia, with early onset dementia, and with physical disabilities  **F**: Due to increased use of phone counselling, problems were identified at an early stage, and the need for face-to-face counselling gradually decreased  **ExtCond**: Programme was based on long-term activities by public health nurses in a rural town and evolved over three cycles at individual,  group, community level (cooking programme: to regain procedural skills; group activities: to increase interactions with family members and with other persons with dementia;  culturally relevant sequential activities: to foster community participation) |
| Salfi^b^ nonanonym  [85-87] | Technology-based counselling as a part of a comprehensive programme with non-technology-based components | Providing telephone support as follow-up to their other support programmes, caregiver- or provider-initiated | N/A | N/A | N/A | Registered nurses with dementia training | Family caregivers | N/A  Based on need  N/A | **ImS**: N/A  **M**: N/A  **B**: Loss of context associated with a telephone interaction  **F**: Convenience of telephone support; Counsellors were aware of callers’ situation  **ExtCond**: Part of a caregiver support programme, which included the Adult Day Program, support groups, educational sessions, and telephone support |

*Abbreviations*: ADS: Alzheimer's Disease Society; B: Implementation barriers; CANDID: Counselling and Diagnosis in Dementia; COACH: Comprehensive Older Adult and Caregiver Help; D-PACT: Dementia - PersonAlised Care Team; ExtCon: External conditions; F: implementation facilitators; FITT-C: Family Intervention: Telephone Tracking – Caregiver; FITT-D: Family Intervention: Telephone Tracking – Dementia; Helpline Alz Ass East Massa: Helpline of the Alzheimer's Association of Eastern Massachusetts; ICSS: Internet-based Caregiver Support Service; ImS: Implementation strategy; min: minutes; M: Modifications of interventions; N/A: not applicable/not available; NCSP: National Call Support Program for Alzheimer’s patients and their caregivers; NVAMP: Nurse Video With Assisted Modeling Program; ODCC: Okayama Dementia Call Center; RCT: Randomized controlled trial; RCTM: Residential Care Transition Module; RLTC: residential long-term care; STAV: Support for family caregivers (“STöd för AnhörigVårdare”); TeleFAMILIES: telehealth-administered Families Access to Memory Impairment and Loss Information, Engagement, and Supports

*Notes*:  ^a^ Fields marked in gray have been published previously [8].

^b^ When no name is reported, the name of the first author was assigned to the intervention.

^d^ An additional booster call was made within a month; the exact time was not specified.
